# Expression of placental CD146 is dysregulated by prenatal alcohol exposure and contributes in cortical vasculature development and positioning of vessel-associated oligodendrocytes

**DOI:** 10.3389/fncel.2023.1294746

**Published:** 2024-01-10

**Authors:** Camille Sautreuil, Maryline Lecointre, Jessica Dalmasso, Alexis Lebon, Matthieu Leuillier, François Janin, Matthieu Lecuyer, Soumeya Bekri, Stéphane Marret, Annie Laquerrière, Carole Brasse-Lagnel, Sophie Gil, Bruno J. Gonzalez

**Affiliations:** ^1^Rouen Université, Inserm U1245 – Team “Epigenetics and Pathophysiology of Neurodevelopmental Disorders”, Normandie Université, Normandy Centre for Genomic and Personalized Medicine, Rouen, France; ^2^Université de Paris, INSERM, UMR-S 1139, 3PHM, Paris, France; ^3^Rouen Université, US51 HeRacLeS, PRIMACEN Platform, Faculty of Biological Sciences, Normandie Université, Mont-Saint-Aignan, France; ^4^Rouen Université, Inserm U1096, Rouen, France; ^5^Rouen Université, CHU Rouen, Department of Metabolic Biochemistry, Normandie University, Rouen, France; ^6^Rouen Université, CHU Rouen, Department of Neonatal Pediatrics and Intensive Care, Rouen, France; ^7^Rouen Université, CHU Rouen, Department of Pathology, Rouen Normandy Hospital, Rouen, France

**Keywords:** fetal alcohol syndrome, angiogenesis, neurovascular development, neuroplacentology, biomarker, diagnosis

## Abstract

Recent data showed that prenatal alcohol exposure (PAE) impairs the “placenta–brain” axis controlling fetal brain angiogenesis in human and preclinical models. Placental growth factor (PlGF) has been identified as a proangiogenic messenger between these two organs. CD146, a partner of the VEGFR-1/2 signalosome, is involved in placental angiogenesis and exists as a soluble circulating form. The aim of the present study was to investigate whether placental CD146 may contribute to brain vascular defects described in fetal alcohol spectrum disorder. At a physiological level, quantitative reverse transcription polymerase chain reaction experiments performed in human placenta showed that CD146 is expressed in developing villi and that membrane and soluble forms of CD146 are differentially expressed from the first trimester to term. In the mouse placenta, a similar expression pattern of CD146 was found. CD146 immunoreactivity was detected in the labyrinth zone and colocalized with CD31-positive endothelial cells. Significant amounts of soluble CD146 were quantified by ELISA in fetal blood, and the levels decreased after birth. In the fetal brain, the membrane form of CD146 was the majority and colocalized with microvessels. At a pathophysiological level, PAE induced marked dysregulation of CD146 expression. The soluble form of CD146 decreased in both placenta and fetal blood, whereas it increased in the fetal brain. Similarly, the expression of several members of the CD146 signalosome, such as VEGFR2 and PSEN, was differentially impaired between the two organs by PAE. At a functional level, targeted repression of placental CD146 by *in utero* electroporation (IUE) of CRISPR/Cas9 lentiviral plasmids resulted in (i) a decrease in cortical vessel density, (ii) a loss of radial vascular organization, and (iii) a reduced density of oligodendrocytes. Statistical analysis showed that the more the vasculature was impaired, the more the cortical oligodendrocyte density was reduced. Altogether, these data support that placental CD146 contributes to the proangiogenic “placenta–brain” axis and that placental CD146 dysfunction contributes to the cortical oligo-vascular development. Soluble CD146 would represent a promising placental biomarker candidate representative of alcohol-induced neurovascular defects in neonates, as recently suggested by PlGF (patents WO2016207253 and WO2018100143).

## Introduction

Fetal alcohol syndrome (FAS) is the most severe manifestation of fetal alcohol spectrum disorder (FASD). It is associated with several clinical defects, such as craniofacial dysmorphism, intrauterine growth retardation and neurodevelopmental defects, leading to behavioral deficits (Riley et al., 2003, 2011). Based on these criteria, an early diagnosis of FAS is possible. Nevertheless, most children with FASD do not exhibit the characteristic physical features of FAS; however, they are not devoid of neurobehavioral disabilities (attention deficits and hyperactivity), which remain undetected until they are school age (Chasnoff et al., 2015). Consequently, numerous infants with FASD are diagnosed late or misdiagnosed, and the challenge for clinicians consists of the early diagnosis of FASD to intervene appropriately and save several years of care.

During pregnancy, the placenta is an ephemeral organ that provides oxygen and nutrients to the fetus and contributes to the clearance of catabolites released by the fetus. In addition to its key role in the control of fetal metabolism, the placenta also exerts important endocrine and immunomodulation functions that are vital for the establishment and continuation of pregnancy. For example, secretions of placental growth hormone (PGH) or human placental lactogen (hPL) are pivotal for fetal growth (Alsat et al., 1998). Moreover, the placenta is a major source of proangiogenic factors from the VEGF family, such as placental growth factor (PlGF; Cao et al., 1997). Several reports described an impact of alcohol consumption during pregnancy on PlGF expression (Haghighi Poodeh et al., 2012; Lui et al., 2014). In particular, using a transcriptomic approach, Savage and coworkers showed that PlGF expression is reduced after moderate alcohol exposure (Rosenberg et al., 2010), while another study reported that prenatal alcohol exposure (PAE) during the last gestational week (GW) in mice resulted in a decrease in PlGF levels correlated with a marked disorganization of cortical vessels in the fetal brain (Lecuyer et al., 2017). Altogether, these data raised the hypothesis of a functional “placenta–brain” axis, which could be involved in the control of fetal brain angiogenesis. Consistent with this hypothesis, it has been recently shown that targeted repression of PlGF expression in the placenta mimicked the effects of PAE on cortical vasculature, whereas placental overexpression of PlGF rescued the effects of alcohol on brain microvessels (Lecuyer et al., 2017).

Cluster of differentiation 146 (CD146) is a glycoprotein belonging to the immunoglobulin superfamily (Lehmann et al., 1989). In endothelial cells, two isoforms of membrane CD146 (mCD146) generated by alternative splicing have been identified (Lehmann et al., 1989; Taira et al., 1995). mCD146 isoforms display angiogenic properties. In particular, they are involved in the stabilization of neovessel junctions and in the regulation of the PlGF/VEGF signalosome (Jiang et al., 2012; Stalin et al., 2013). Indeed, mCD146 constitutes with angiomotin, VEGF-R1, VEGF-R2, and presenilin-1 (PSEN-1) a complex molecular scaffold network in endothelial cells (Stalin et al., 2016). In addition, mCD146 can be cleaved by metalloproteinases, leading to a soluble and circulating form of CD146 (sCD146; Bardin et al., 1998; Boneberg et al., 2009). At a pathological level, sCD146 has been described as a marker of tumor growth and metastasis in humans, and high levels of circulating sCD146 are associated with a poor prognosis (Sers et al., 1994; An et al., 2020). However, the involvement of membrane and soluble forms of CD146 in physiological angiogenesis remains to be highlighted. For example, a translational study conducted in 50 pregnant women revealed that sCD146 is physiologically decreased in pregnant women, while in a rat model, repeated systemic injections of sCD146 after mating caused a significant decrease in the pregnancy rate and the number of embryos (Kaspi et al., 2013).

In addition to PlGF, the placental endothelium strongly expresses CD146 (Shih, 1994), and in light of the literature, we hypothesized that (i) sCD146 would be present in the fetal blood, (ii) the expression of mCD146 and members of its signalosome would be impaired by *in utero* alcohol exposure, and (iii) placental CD146 may contribute to the cortical angiogenesis of the fetus. To test these working hypotheses, we first intended to validate both membrane and soluble forms of CD146 expression in the human placenta before moving to a mouse model to compare the CD146 expression profiles in the placenta, fetal blood, and fetal brain. Then, we investigated the effects of PAE on the expression of CD146 and different members of the PlGF/VEGF signalosome in both the placenta and fetal brain. Finally, we performed a functional study consisting of characterizing the effects of targeted placental repression of CD146 on the development of the fetal brain vasculature.

## Materials and methods

### Chemicals

Hoechst 33258, povidone iodine, bovine serum albumin (BSA) and protease inhibitor cocktail were obtained from Sigma-Aldrich (Saint-Quentin-Fallavier, France). The characteristics of the antibodies raised against angiomotin, CD31, CD146, GFP, Olig2, presenilin-1, VEGF-R1, VEGF-R2, vinculin, and β-actin are detailed in Supplementary Table 1. Two membrane isoforms of CD146 with similar molecular weights have been characterized and differ from their intracellular portions (Leroyer et al., 2019). The CD146 antibodies used in the present study do not discriminate these two isoforms. The goat anti-rabbit IgG-HRP (sc-2030), goat anti-mouse IgG-HRP (sc-2031), donkey anti-goat IgG-HRP (sc-2033), and CD146-CRISPR/Cas9 KO Plasmid (Mel-CAM plasmid CRISPR, sc-429966) used for *in utero* electroporation (IUE) were obtained from Santa Cruz Biotechnology (Santa Cruz, CA, USA). Alexa Fluor^®^ 594 donkey anti-rabbit IgG (A-21207), Alexa Fluor^®^ 594 Donkey anti-rat IgG (A-21209), Alexa Fluor^®^488 Chicken anti-mouse IgG (A-21200), and Alexa Fluor^®^ 488 anti-rat IgG (A-21470), used for immunohistochemistry, were from Invitrogen (Eragny, France). The mouse sCD146 ELISA kit was purchased from Elabscience^®^ (TX, USA). Isoflurane was purchased from Baxter (Maurepas, France). The lysis buffer was from Cell Signaling Technology (Danvers, MA, USA).

### Human placenta collection

Placenta were obtained from Port-Royal Maternity, the Mutualist Institute Montsouris, the Private Hospital of Antony, the Antoine-Béclère Hospital, and the Beaujon Hospital after obtaining written consent from informed patients and approval from our local ethics committee (CPP: 2015-May-13909). Placenta were collected up to 1 h after surgery from healthy women at term pregnancies (40 GW) by cesarean sections and at the first trimester (8 and 13 GW). Chorionic villi were collected under sterile conditions and washed in calcium- and magnesium-free Hanks’ Balanced Salt solution 1× (Gibco #14175, ThermoFisher Scientific^®^, Illkirch-Graffenstaden, France). For transcriptional analyses, villi were incubated overnight with RNA Later^®^ (Qiagen^®^, Courtaboeuf, France), frozen in liquid nitrogen and stored at −80°C until RNA extraction. For protein analysis, villi were frozen directly in liquid nitrogen and stored at −80°C for protein extraction.

### Animals and *in vivo* treatments

National Marine Research Institute mice from Janvier (Le Genest-Saint-Isle, France) were used according to the French Ethical Committee recommendations and European directives 2010/63/UE. Experiments were performed under the supervision of authorized investigators (authorization no. APAFIS#22136-2019092013438607 v4 from the Ministère de l’Enseignement Supérieur, de la Recherche et de l’Innovation). From gestational day (GD) 15 to GD 20, pregnant mice received a daily subcutaneous injection of sodium chloride (NaCl 0.9%) or alcohol (3 g/kg, Fisher Scientific) diluted in NaCl (50%, v/v). For prenatal stages (GD15, GD17, and GD20), placentas and associated fetal brains were collected. For postnatal stages (P2, P5, P10, P15, and P20), only brains were harvested. For the quantitative reverse transcription polymerase chain reaction (qRT-PCR) and Western blot analyses, placentas and cortices were immediately frozen on dry ice and stored at −80°C. For the histological studies, placenta and brains were immersed in 0.1 M phosphate-buffered saline (PBS) containing 4% paraformaldehyde for 24 h at 4°C. Then, they were incubated overnight in 30% sucrose and frozen in isopentane (−40°C). Frontal sections (25 μm thick) were cut on a cryomicrotome (Leica Microsystems, Nanterre, France) and stored at −20°C until used. Females and males were identified. Since results showed no sex differences (Supplementary Figure 1), data were pooled on the same figure as previously done (Léger et al., 2020a; Brosolo et al., 2022).

### RNA extraction, reverse transcription, and quantitative real-time PCR in humans

Chorionic villi previously incubated with RNAlater and stored at −80°C were lysed with lysis buffer from an RNeasy^®^ Mini Kit (Qiagen^®^). Total RNA was then extracted following the manufacturer’s instructions using the RNeasy^®^ Mini Kit and DNase from Qiagen^®^. Afterward, total RNA was quantified using a Nanodrop© spectrophotometer (ND-1000), and RNase inhibitor (RNAseOUT Invitrogen©) was added to each sample. All samples were kept at −80°C until reverse transcription. Reverse transcription was performed for 500 ng RNA with the Superscript III First Strand Synthesis system and the Random primer and dNTP mix (Invitrogen^®^, Eragny, France) using the PCR system 2700 thermocycler (Applied Biosystems^®^ GeneAmp^®^, Villebon sur Yvette, France). Quantitative real-time PCR (40 cycles) was performed in a 10 μl reaction volume in the presence of Takyon TmROX SYBR Master Mix dTTP Blue^®^ (Eurogentec^®^, Angers, France) using a Thermocycler 7900 HT Fast Real-Time PCR System (Applied Biosystems^®^). The threshold cycle (Ct) was measured as the number of cycles at which the reporter fluorescent emission first exceeded the background. The relative amounts of mRNA were estimated using the ΔΔCt method and then expressed as fold change. Primers for RPLO and RPL13 were used for the normalization of the results obtained with placental villi. The results were analyzed on SDS 2.4© and GraphPad Prism© software.

### Western blot analysis of chorionic villi in humans

Total protein extracts from villi were obtained by extraction with NE-PER reagents (Thermo Fisher Scientific^®^, Les Ulis, France) that allow solubilization and separation for cytoplasmic and nuclear proteins. Thereafter, protein concentrations were determined using the Pierce™ BCA Protein Assay Kit (Thermo Fisher Scientific^®^). Equal amounts of proteins (40 μg) were separated on 4%–15% SDS–PAGE mini-PROTEAN^®^ TGX^®^ precast protein gels under reducing conditions and transferred onto a nitrocellulose membrane (TransBlot Turbo transfer pack^®^, Bio-Rad^®^, Marnes-la-Coquette, France). Blots were incubated overnight with the primary antibody at 4°C and then for 2 h with the appropriate Fluor-conjugated secondary antibody (Thermo Scientific^®^, Sigma^®^). Details on the primary antibodies are provided in Supplementary Table 1. Blots were scanned with an Odyssey^®^ Imaging System (Li-COR©, Lincoln, NE, USA). Quantification was performed using Li-COR Odyssey and Excel© (Microsoft, Redmond, WA, USA) software.

### RNA extraction and qRT-PCR in mouse tissues

Placenta from control and alcohol-exposed mice and the associated brain cortices from fetuses were harvested for total RNA extraction using NucleoSpin^®^ RNA Plus (Macherey-Nagel, Hoerdt, France) according to the manufacturer’s recommendations. RNA was reverse transcribed to cDNA using a Reverse Transcription System kit (Promega, Madison, WI, USA) with reverse transcription buffer, MgCl_2_, dNTPs, and avian myeloblastosis virus reverse transcriptase (AMVRT). PCR (40 cycles) was performed using SYBR Green Supermix (Bio-Rad, Marnes-la-Coquette, France). The efficiency of PCR amplification was assessed for each primer set with the slope of a standard curve generated with serial dilutions of placental and cortical brain cDNA, which was close to 3.3. Quantitative RT-PCR was performed using the gene-specific forward and reverse primers described in Supplementary Table 2. For each sample, the purity of the PCR product was controlled with dissociation curves, and the cDNA amount was calculated using the ΔΔCt method. Glyceraldehyde 3-phosphate dehydrogenase (GAPDH) was used as an internal control. The expression of GAPDH mRNA was not affected by ethanol exposure.

### Western blot analysis of placental and cortical extracts in mice

Placentas and cortices from fetuses and neonates were homogenized in 250 μl of lysis buffer (Cell Signaling Technology). The homogenates were centrifuged (18,000 *g*; 20 min), and then the supernatants were used for Western blotting. Fifty micrograms of protein extracts from cortical and placental samples were suspended in Laemmli buffer (100 mM HEPES; pH 6.8; 10% β-mercaptoethanol; 20% SDS) and boiled for 5 min. Then, they were loaded on a 10% SDS-polyacrylamide gel. After electrophoresis, proteins were transferred to a nitrocellulose membrane. The membrane was incubated with different blocking solutions [1× Tris buffer saline (TBS); 0.05% Tween-20; 5% non-fat milk or 1× TBS; 0.05% Tween-20; 5% BSA] at room temperature for 1.5 h and incubated overnight with primary antibodies (Supplementary Table 1). After incubation with the corresponding secondary antibodies coupled to peroxidase (Santa Cruz Biotechnology, Santa Cruz, CA, USA), proteins were visualized using an enhanced chemiluminescence ECL Plus immunoblotting detection system (Amersham Biosciences Europe GmbH, Freiburg, Germany). The intensity of the immunoreactive bands was quantified using a blot analysis system (Bio-Rad Laboratories, Marne la Coquette, France), and β-actin was used as a loading control. Commercial markers (SeeBlue Pre-stained Standard, Invitrogen) were used as molecular weight standards.

### Immunohistochemistry in mice

Placenta and brain slices previously fixed with 4% PFA in PBS were incubated overnight at 4°C with various primary antibodies (Supplementary Table 1) diluted in incubation buffer (PBS containing 1% BSA and 3% Triton X-100). Next, the slices were rinsed twice with PBS for 20 min and incubated in the same buffer containing the appropriate secondary antibody. Cell nuclei were visualized by incubating the slices for 5 min with 1 g/L Hoechst 33258 in PBS. Fluorescent signals were observed with a Leica DMI 6000B microscope (Leica Microsystems). The specificity of the immunoreaction was controlled by omitting the primary antibody.

### Quantification of soluble CD146 levels in fetal blood by ELISA

Control and alcohol-exposed fetuses were collected at GD20 using the same surgical protocol as for electroporations. Afterward, E20 fetuses were rinsed in ice-cold PBS to remove excess blood thoroughly and rapidly killed by decapitation. Cephalic blood from fetuses was collected using heparinized capillary tubes and pooled, and after centrifugation (1,500 × *g* for 10 min), plasma was frozen until the assay. ELISA was then performed using the instructions provided in the commercial kit (Elabscience^®^, TX, USA).

### *In utero* placental transfection of plasmid vectors and repression of CD146 expression

Pregnant mice at GD13 were anesthetized with isoflurane using an anesthetic vapourizer for a maximum of 40 min (MiniHUB V2.1, TemSega, Pessac, France). A laparotomy was performed to allow access to uterine horns. The abdominal cavity, especially the exposed uterine horn, was kept moist with warmed physiological solution. During surgery, the body temperature of the mouse was controlled and maintained using a hotplate (Homeothermic Monitoring System, Harvard Apparatus, MA, USA). Injections of the CD146-CRISPR/Cas9 KO and pCAG-GFP (PCIG2-IRES-GFP) plasmids were performed using micropipettes made of glass capillaries (0.58 mm inner diameter, 1.0 mm outer diameter, Harvard Apparatus, UK) with a P-97 flaming/brown micropipette puller (Sutter Instrument Company; Novato, CA, USA). CD146 CRISPR/Cas9 KO plasmids consisted of a pool of three target-specific lentiviral vectors each encoding the Cas9 nuclease and a guide RNA targeting 20 nucleotides designed to knock down gene expression. To define the exact injected volume, a millimeter scale was fixed on the capillary and calibrated with defined volumes. The injection depth within the placenta was 0.5 mm, and 3 μl of the solution was injected. For electroporation, the appropriate voltage was applied *via* specialized platinum electrodes Nepagene CUY 650P5 (Nepagene Co., Ichikawa, Japan) with the following parameters: interval cycle length 30 ms, interval pause 450 ms. The voltage conditions were controlled on the NEPA21 type II Electroporator (Nepagene Co., Ichikawa, Japan). After electroporation, the uterine horn was carefully replaced in the abdominal cavity, and the abdominal walls were sutured with sterile Silk Suture Prolene 6-0, MPP2832 (ETHICON, Lindingö, Sweden). Mice were kept at a warm temperature on a hot plate until total recovery from anesthesia. Fetal brains corresponding to *in utero*-transfected placenta were collected at E20 for the CD146 Western blot experiments and vessel morphometric analysis.

### Labeling and quantification of the cortical microvascular network in mouse fetuses

To visualize the brain microvascular network on histological sections from control and alcohol-exposed animals, immunohistochemistry targeting the endothelial cell marker CD31 was performed. Immunolabeling was analyzed under a DMI 6000 fluorescence microscope (Leica Microsystems) equipped with a CCD camera (Roper Scientific, Lisses, France). For vascular network measurements, a ratiometric approach was employed using Metamorph software (Roper Scientific). Images of histological slices were acquired under standardized conditions (magnification and brightness) and saved in TIFF format using the computer-assisted image analysis station from Roper Scientific. For each slice, the angular orientation of microvessels was quantified using Metamorph software (Roper Scientific). Quantification was performed in the sensorimotor cortex, and 6 fetuses from three independent pregnant mice were analyzed per group. A frame of lines was defined perpendicular to the cortical border for each section. For microvessels parallel to these lines, the Metamorph software arbitrarily attributed the angular value of 0°. The maximal angular value was 90°. Measurement of the vessel density was performed within the whole thickness of the sensorimotor cortex or by distinguishing the superficial cortical layers (SL), the deep cortical layers (DL), and the corpus callosum (CC). A threshold was set in order to distinguish the CD31-positive structures from the background and the corresponding regions of interest (ROI) were selected by segmentation. For each ROI, the ratio “vascular area/cortical area” was calculated by the Metamorph software.

### Quantification of the Olig2-positive cell density in the developing cortical layers and corpus callosum

Measurement of the oligodendrocyte density in cortical layers and CC was performed after immunostaining of E20 brain slices using the Olig2 antibody (Supplementary Table 1). Images were acquired at 10× magnification using a Leica Thunder Imaging System CTR5500, and ROIs were defined within the whole thickness of the sensorimotor cortex or by distinguishing the three regions SL, DL, and CC. Fluorometric analysis using the multi-point counting tool of the Fiji software gave access to the number of immunoreactive cells present in the ROI. Density was then determined by a ratio between the number of cells and the ROI area. The analysis was repeated in both hemispheres and in three slices per animal (Brosolo et al., 2022).

### Quantification of the oligo-vascular interactions in the developing cortex of control and CD146-repressed fetuses

The quantification of vessel-associated oligodendrocytes was performed in mouse brain slices at E20 after double immunostaining with Olig2 (oligodendrocytic lineage) and CD31 (endothelial cells) antibodies (Supplementary Table 1). Z stacks acquisitions were done at 40× magnification using a Leica Thunder Imaging System CTR5500 in three groups: non-electroporated placenta (Ctrl), CRISPR-negative/GFP-positive electroporated placenta (Ctrl_ep_), and CD146-CRISPR/Cas9/GFP transfected placenta (CD146-CRISPR). Afterward, Z-stack series of images were loaded into IMARIS imaging software 9.0.2 (Bitplane, Zurich, Switzerland) for 3D reconstruction (Supplementary Videos 1, 2). To validate a vessel-oligodendrocytes interaction, the maximal distance between the center part of Olig2-positive cells and the center part of the vessel was fixed at 10 μm (Brosolo et al., 2022).

### Statistical analyses

Statistical analysis was performed using the biostatistics software Prism (GraphPad Software, La Jolla, CA, USA). The tests used for each experiment, the number of independent experiments and the *p*-values are summarized in Supplementary Table 3.

## Results

### Expression of CD146 and members of its signalosome in human placenta

**FIGURE 1:**
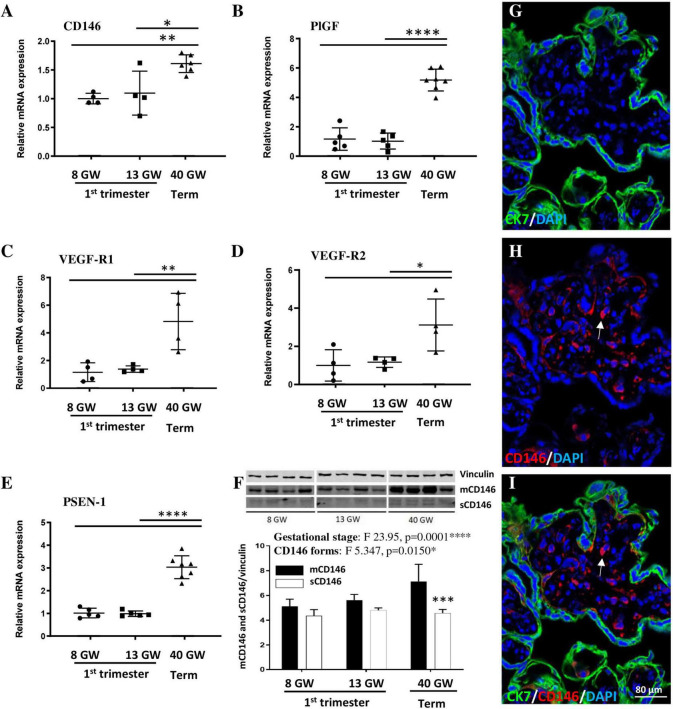
Expression of CD146, PlGF, and members of the signalosome in the human placenta at three gestational stages. Comparison of the relative expression of CD146 **(A)**, PlGF **(B)**, VEGF-R1 **(C)**, VEGF-R2 **(D)**, and PSEN-1 **(E)** by qRT-PCR in villous extracts at 8, 13, and 40 GW. **(F)** Quantification by Western blotting of mCD146 and sCD146 protein levels in villous extracts from human placentas at 8, 13, and 40 GW. Immunohistochemistry experiments visualizing trophoblasts (CK7 antibody; **G,I**), CD146 (arrows; **H,I**), and nuclei (DAPI; **G–I**) in a 40 GW placenta. *p < 0.05; **p < 0.01; ***p < 0.001; ****p < 0.0001 vs. as indicated in the graphs. Each value is the mean (± SEM). Statistical details are provided in Supplementary Table 3.

To explore the expression levels of CD146 in the developing placenta, villous extracts were prepared at three gestational stages (8, 13, and 40 GW; Figure 1). qRT-PCR experiments showed that the expression of CD146 mRNA was similar between GW8 and GW13 (Figure 1A). In contrast, CD146 mRNA levels significantly increased from the first trimester of pregnancy to term (*p* < 0.05; *p* < 0.01; Figure 1A). A similar expression pattern was found for other members of the CD146 signalosome, including PlGF (*p* < 0.0001; Figure 1B), VEGF-R1 (*p* < 0.01; Figure 1C), VEGF-R2 (*p* < 0.05; Figure 1D), and PSEN-1 (*p* < 0.0001; Figure 1E). To discriminate between the membrane and soluble forms of CD146, Western blot experiments were conducted (Figure 1F). Two-way ANOVA analysis showed a significant gestational age interaction (*F* 23.95; ****p = 0.0001) and CD146 forms interaction (*F* 5.347; *p = 0.0150). Post-test analysis showed a significant difference between membrane and soluble CD146 forms at GW40 (*p* < 0.001; Figure 1F). Immunohistochemistry experiments showed a CD146 expression localized in intravillous vessels (Figures 1G–I; arrows).

### Expression of mCD146 and sCD146 in mouse placenta

**FIGURE 2:**
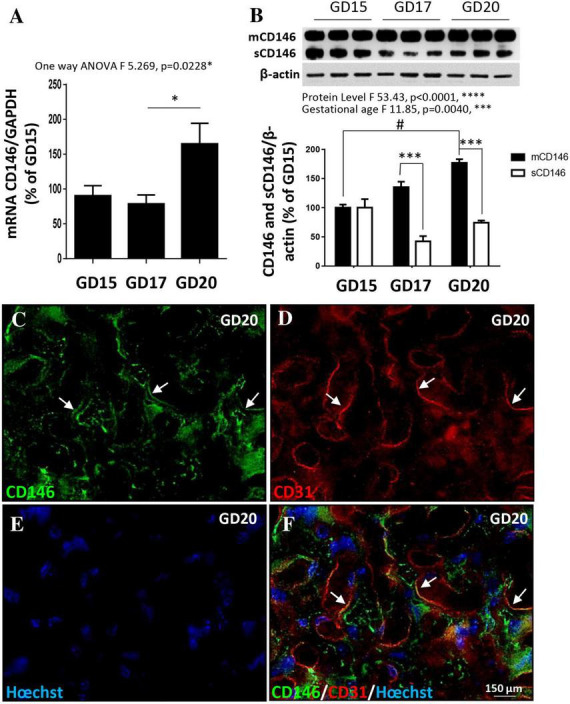
Expression of CD146 in the mouse placenta at three gestational stages. **(A)** Comparison of the relative expression of CD146 by qRT-PCR in mouse placental extracts at GD15, GD17, and GD20. **(B)** Quantification by Western blotting of mCD146 and sCD146 protein levels at GD15, GD17, and GD20. A representative immunoblot is shown on the top of the graph. **(C–F)** Immunohistochemistry experiments showing the localization of CD146 expression in the placenta at GD20. CD146 immunoreactivity is detected in the labyrinth zone **(C)** and colocalizes with the endothelial cell marker CD31 (arrows; **D,F**). Hoechst was used to label nuclei **(E,F)**. Each value is the mean (± SEM). ^#^*p* < 0.05 vs. GD15; ***p < 0.001 vs. mCD146. *p < 0.05 vs. GD17 and ****p < 0.0001 from grouped ANOVA analysis. Statistical details are provided in Supplementary Table 3.

Whereas CD146 has been shown to contribute to the control of trophoblast migration and placental vascular development (Kaspi et al., 2013), its expression pattern in mouse placenta has been scarcely investigated. CD146 expression was explored by qRT-PCR and Western blot experiments at three gestational stages (GD15, GD17, and GD20; Figure 2). CD146 mRNA was detected beginning on GD15, one-way ANOVA analysis showed that mRNA expression statistically increased between ages (*F* 5.269; *p* = 0.0228) and post-test analysis showed a significant difference between GD17 and GD20 (*p* < 0.05; Figure 2A). At the protein level, the Western blot experiments showed that the membrane form of CD146 progressively increased from GD15 to GD20 (Figure 2B). Two-way ANOVA analysis showed a significant age interaction (*F* 11.85; ***p = 0.004). The soluble form of CD146 was detected after GD15 (Figure 2B). No difference between mCD146 and sCD146 levels was found at this stage (Figure 2B). At GD17 and GD20, the soluble form of CD146 was significantly lower than the membrane form (*p* < 0.001; Figure 2B). Immunohistochemistry experiments indicated that CD146 immunoreactivity was detected in the labyrinth zone of the placenta at GD20 (Figure 2C) and colocalized with CD31-positive cells, a marker of endothelial cells (Figures 2D–F; arrows).

### Expression of mCD146 and sCD146 in the fetal mouse brain

**FIGURE 3:**
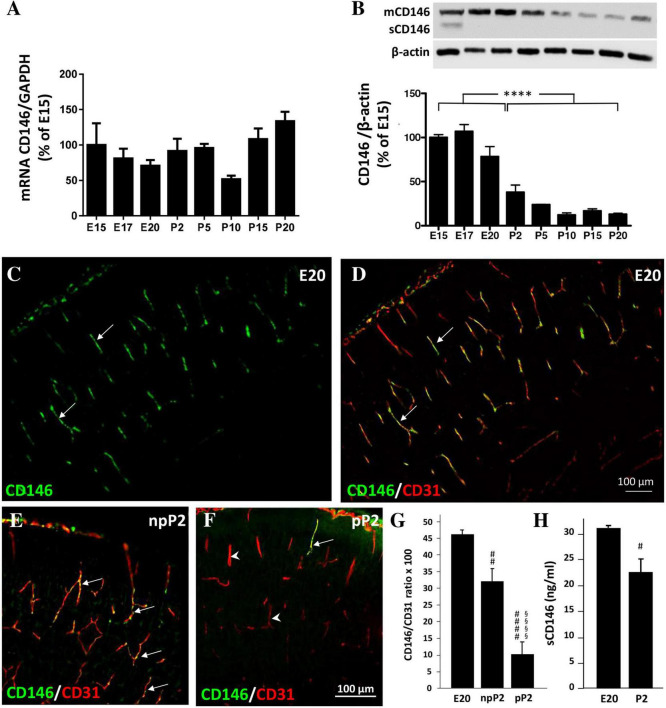
Expression of CD146 in the developing cortex. **(A)** Comparison of the relative expression of CD146 by qRT-PCR in the developing cortex at pre- and postnatal stages ranging from E15 to P20. **(B)** Quantification by Western blotting of mCD146 and sCD146 protein levels at pre- and postnatal stages ranging from E15 to P20. A representative immunoblot is shown on the top of the graph. **(C,D)** Visualization by immunohistochemistry of CD146-positive labeling in the developing cortex at E20 **(C)**. Note that CD146 immunoreactivity colocalizes with radial microvessels labeled with the endothelial cell marker CD31 (**D**; arrows). Comparison of CD146 immunolabeling in P2 cortices without (npP2; **E**) or after intracardiac perfusion with PBS (pP2; **F**). Note the decrease of the vessel-associated CD146 immunolabeling (**F**; arrows/arrowheads). **(G)** Quantification of the CD146/CD31 intensity ratio in radial cortical microvessels from mouse fetuses (E20) and in P2 cortices without (npP2) or after intracardiac perfusion (pP2). Note that in cortices of perfused P2 neonates the CD146/CD31 intensity ratio is decreased. **(H)** Quantification by ELISA of soluble CD146 in fetal blood at E20 and P2. Each value represents the mean (± SEM). ****p < 0.0001 vs. the fetal group; ^#^*p* < 0.05, ^##^*p* < 0.01, ^####^*p* < 0.0001 vs. E20; ^§§§§^ *p* < 0.0001 vs. npP2. Statistical details are provided in Supplementary Table 3.

As done for the placenta, the expression of CD146 was investigated by qRT-PCR and Western blotting in the developing cortex at the same prenatal stages [embryonic day 15 (E15), E17, and E20; Figure 3]. Experiments were continued after birth from postnatal day 2 (P2) to P20 (Figure 3). CD146 mRNAs were detected as early as E15 and no significant difference was found between stages (Figure 3A). Regarding the protein, CD146 expression in the developing cortex was markedly regulated (Figure 3B). In particular, a grouped analysis showed a marked decrease in the membrane form of CD146 between the fetal and postnatal stages (*p* < 0.0001; Figure 3B). Interestingly, in contrast with the placenta, in the developing brain, the soluble form of CD146 was poorly detected and mostly at E15 (Figure 3B). Immunohistochemistry experiments performed at E20 and P2 showed that CD146 immunostaining was restricted to microvessels visualized with CD31 antibodies (Figures 3C, D; arrows). Interestingly, perfusion of mouse brains with PBS at P2 strongly reduced the CD146 immunoreactivity detected in microvessels (Figures 3E, F; arrowheads). Quantification of the CD146 immunoreactivity by thresholding revealed the following: first, a significant decrease in the fluorescent signal between E20 and P2 (*p* < 0.01; Figure 3G); and second, a marked decrease in the CD146 immunoreactivity after blood removal, suggesting that part of the fluorescent signal would consist of circulating sCD146 (*p* < 0.0001; Figure 3G). To test this hypothesis, ELISA experiments targeting the soluble form of CD146 were conducted in the cephalic blood of E20 fetuses and P2 neonates (Figure 3H). The amounts of sCD146 ranged from 20 to 35 ng/ml and significantly decreased from E20 to P2 (*p* < 0.05; Figure 3H).

### Effect of PAE on CD146 levels in the placenta, fetal brain, and cephalic blood

**FIGURE 4:**
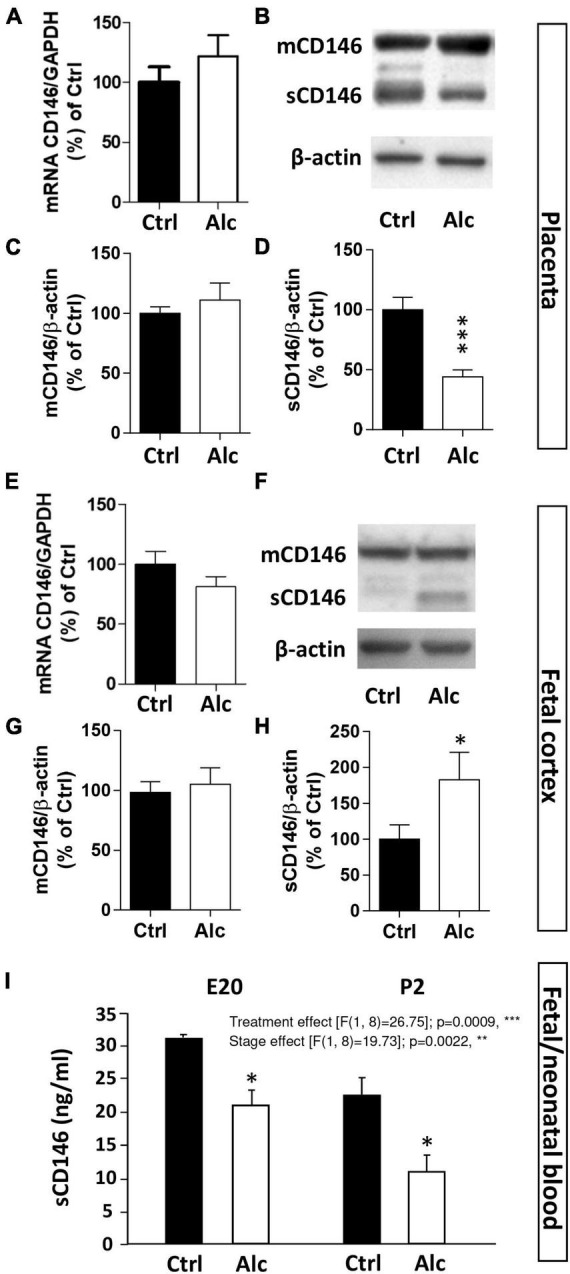
Effect of PAE on placental and cortical expression of CD146. **(A)** Effect of alcohol on CD146 mRNA expression in mouse placental extracts at GD20. Visualization **(B)** and quantification by Western blotting of the effects of PAE on mCD146 **(C)** and sCD146 **(D)** expression in mouse placental extracts at GD20. **(E)** Effect of PAE on CD146 mRNA expression in the cortex of E20 fetuses. Visualization **(F)** and quantification by Western blotting of the effects of PAE on mCD146 **(G)** and sCD146 **(H)** in mouse cortical extracts at E20. **(I)** Effect of PAE on CD146 levels measured by ELISA in the cephalic blood from E20 fetuses and P2 neonates. Each value represents the mean (± SEM). *p < 0.05, **p < 0.01, ***p < 0.001 vs. Ctrl. Statistical details are provided in Supplementary Table 3.

Several studies previously reported that PAE impairs the expression of angiogenic factors from the VEGF/PlGF family in both the placenta and brain (Jégou et al., 2012; Lecuyer et al., 2017). Because CD146 acts as a coreceptor of this proangiogenic system, we investigated whether alcohol would be able to alter CD146 expression (Figure 4). The qRT-PCR experiments revealed that PAE did not modify CD146 mRNA expression in the placenta at GD20 (Figure 4A). Similarly, protein analysis by Western blotting showed that PAE did not affect the expression of the membrane form of CD146 (Figures 4B, C). Conversely, PAE induced a significant decrease in the soluble form of CD146 in the placenta (*p* < 0.001; Figures 4B, D).

Similarly, qRT-PCR and Western blot experiments were conducted at the same fetal stage in the cortex of control and alcohol-exposed fetuses (Figures 4E–H). As observed for the placenta, PAE did not modify CD146 mRNA expression in the fetal cortex at E20 (Figure 4E). Regarding proteins, the cortical expression of mCD146 was not impaired by PAE (Figures 4F, G). In contrast to the placenta, a significant increase in the soluble form of CD146 was quantified in the cortex of E20 fetuses exposed to alcohol (*p* < 0.05; Figures 4F, H).

The effect of PAE on circulating levels of CD146 was also quantified in the cephalic blood of E20 fetuses and P2 neonates by ELISA (Figure 4I). Two-way ANOVA analysis showed a significant gestational age interaction (*F* 16.88; **p = 0.0045) and treatment interaction (*F* 20.06; **p = 0.0029). Post-test analysis showed a significant decrease of sCD146 levels in cephalic blood at E20 and P2 (*p* < 0.05; Figure 4I). Regression analysis performed between mean placental sCD146/mCD146 ratio, mean sCD146 blood levels, and mean cortical sCD146/mCD146 ratio showed that slopes between placental s/mCD146 ratio and blood sCD146 levels were similar between control and alcohol groups (Supplementary Figure 2A). In contrast, when comparing s/mCD146 ratios between placenta and fetal brain, a significant slope inversion was found (*p* < 0.001; Supplementary Figure 2B). Altogether, these results indicate that PAE differently impaired the expression of the soluble form of CD146 in the placenta, cephalic blood, and fetal cortex. The opposite dysregulation occurring between the placenta and fetal cortex suggest a possible compensatory process between these two organs.

### Effect of PAE on the CD146 signalosome in mouse placenta and fetal cortex

**FIGURE 5:**
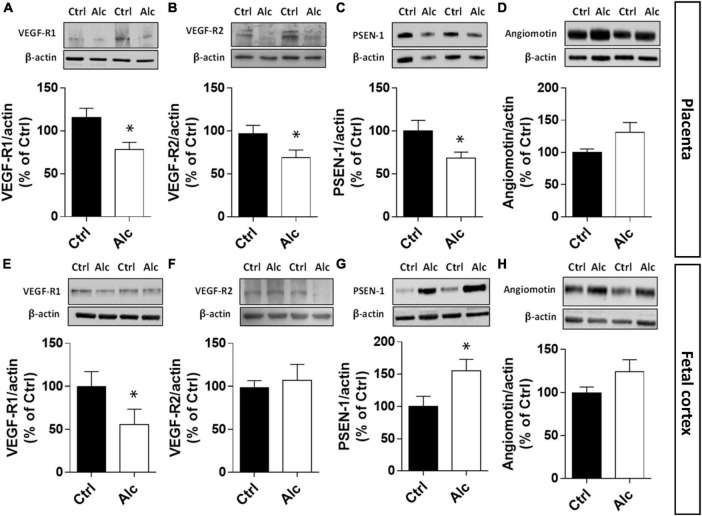
Effect of PAE on the expression of placental and cortical members of the CD146 signalosome. Quantification by Western blotting in the placenta from GD20 pregnant mice of VEGF-R1 **(A)**, VEGF-R2 **(B)**, PSEN-1 **(C)**, and angiomotin **(D)**. Quantification by Western blotting in the cortex from E20 fetuses of VEGF-R1 **(E)**, VEGF-R2 **(F)**, PSEN-1 **(G)**, and angiomotin **(H)**. Each value represents the mean (± SEM). *p < 0.05 vs. control group. Statistical details are provided in Supplementary Table 3.

At a mechanistic level, CD146 has been shown to interact with the VEGF-R1/-R2 signalosome of endothelial cells in both *in vitro* and *in vivo* models (Jiang et al., 2012; Stalin et al., 2013). In particular, soluble CD146 exerts a coreceptor function and interacts with cell signaling proteins such as angiomotin and presenilin-1 (PSEN-1; Stalin et al., 2016). Based on these data from the literature, we investigated the impact of PAE on the VEGF-R1/-R2 signalosome in both placenta and fetal cortex (Figure 5). In the placenta, quantification of VEGF-R1 by Western blotting showed a decreased expression in alcohol-exposed mice at GD20 (*p* < 0.05; Figure 5A). Similarly, a significant decrease in VEGF-R2 expression was found (*p* < 0.01; Figure 5B). Regarding PSEN-1, PAE reduced the expression of this protease (*p* < 0.05; Figure 5C) while no significant effect was found for angiomotin even if its expression tended to increase (Figure 5D). In the developing cortex of age-matched fetuses, a strong reduction in VEGF-R1 occurred at E20 after PAE (*p* < 0.05; Figure 5E), while the expression of VEGF-R2 was not modified (Figure 5F). Unlike the placenta, PSEN-1 expression was strongly increased in the cortex of PAE fetuses (*p* < 0.05; Figures 5C, G) whereas, as found in the placenta, the cortical expression of angiomotin was not significantly affected (Figures 5D, H). Altogether, these results indicate that PAE differently impaired the CD146-associated signalosome in the placenta and fetal brain.

### Effect of a targeted repression of placental CD146 on vasculature of the fetal cortex

**FIGURE 6:**
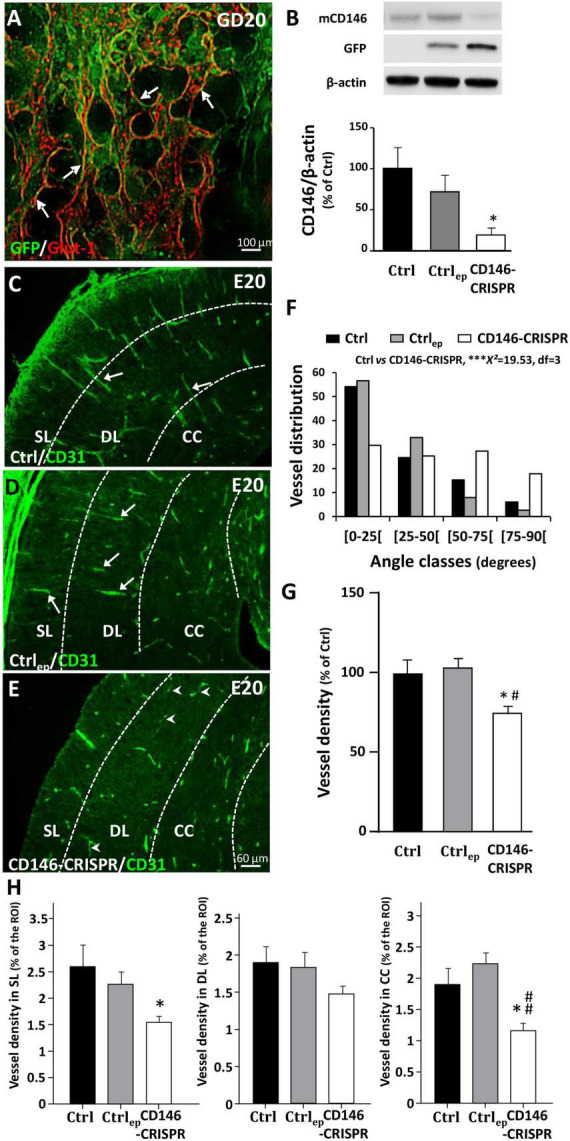
Impact of placental repression of CD146 on the cortical vasculature of E20 fetuses. **(A)** Visualization of control GFP expression after *in utero* placenta electroporation at GD20. **(B)** Quantification by Western blotting of mCD146, GFP, and ß-actin proteins in GD20 placentas from control (no electroporation; Ctrl), electroporated control (control GFP plasmids; Ctrl_ep_), and electroporated CD146-CRISPR (CD146-CRISPR) groups. Representative immunoblots are shown in the top panel of the graph. Visualization of the vasculature in the cortex of fetuses from Ctrl **(C)**, Ctrl_ep_ **(D)**, and CD146-CRISPR **(E)** groups. **(F)** Distribution of cortical microvessel angles in the developing cortex from Ctrl, Ctrl_ep_, and CD146-CRISPR groups. Angle values are distributed in four classes. The angle class [0–25] corresponds to radial microvessels. **(G)** Quantification of the vessel density in the developing cortex of Ctrl, Ctrl_ep_, and CD146-CRISPR groups. **(H)** Quantification of the vessel density in the superficial cortical layers (SL), the deep cortical layers (DL), and the corpus callosum (CC) in the developing cortex from Ctrl, Ctrl_ep_, and CD146-CRISPR groups. Each value represents the mean (± SEM). *p < 0.05 vs. Ctrl, ***p < 0.001, ^#^*p* < 0.05; ^##^*p* < 0.01 vs. Ctrl_ep_. Statistical details are provided in Supplementary Table 3.

Using preclinical and clinical approaches, recent studies have demonstrated a functional “placenta–brain” axis involved in fetal brain angiogenesis (Lecuyer et al., 2017). Because the present data revealed opposite dysregulations of the soluble form of CD146 in the placenta and fetal brain and supported a compensatory process. In order to reinforce a such compensatory mechanism for CD146, we investigated whether CD146 repression in the placenta would affect fetal brain angiogenesis (Figure 6). Invalidation of CD146 was performed using the CRISPR/Cas9 KO strategy coupled to IUE. Visualization of GFP was used to ensure that electroporation occurred in the labyrinth zone of the placenta (Figure 6A; arrows). The efficiency of plasmid transfection and CD146 repression was controlled by Western blotting targeting GFP and mCD146 proteins, respectively (Figure 6B). Electroporation of the placenta with CRISPR-negative/GFP-positive plasmids did not significantly impact mCD146 expression (Ctrl_ep_; Figure 6B). In contrast, transfection of CD146-CRISPR/Cas9 KO plasmids resulted in a significant reduction in the membrane form of CD146 in the placenta (CD146-CRISPR; *p* < 0.05; Figure 6B). Visualization of the cortical vasculature in the E20 fetuses matching placenta was performed by CD31 immunohistochemistry (Figures 6C–E). In both Ctrl and Ctrl_ep_ groups, microvessels had a preferential radial organization in the developing neocortex (Figures 6C, D; arrows). In contrast, in fetuses from the CD146-CRISPR group, major vascular impairments occurred (Figure 6E). Numerous microvessels had a tangential orientation (Figure 6E; arrowheads) and a morphometric quantification of vessel angles showed that placental repression of CD146 resulted in a huge decrease in the proportion of microvessels with radial positioning (***χ^2^ = 19.92, df = 3; Figure 6F). Moreover, the density of microvessels in the developing sensorimotor cortex was significantly reduced when compared with the Ctrl (*p* < 0.0001) and Ctrl_ep_ (*p* < 0.001) groups (Figure 6G). A quantitative analysis considering the SL (Figure 6H, left panel), the DL (Figure 6H, middle panel), and the CC (Figure 6H, right panel) indicated that the placental repression of CD146 significantly reduced the vasculature density in the SL and CC (*p* < 0.05; Figure 6H).

### Effect of a targeted repression of placental CD146 on the cortical positioning of oligodendrocytes

**FIGURE 7:**
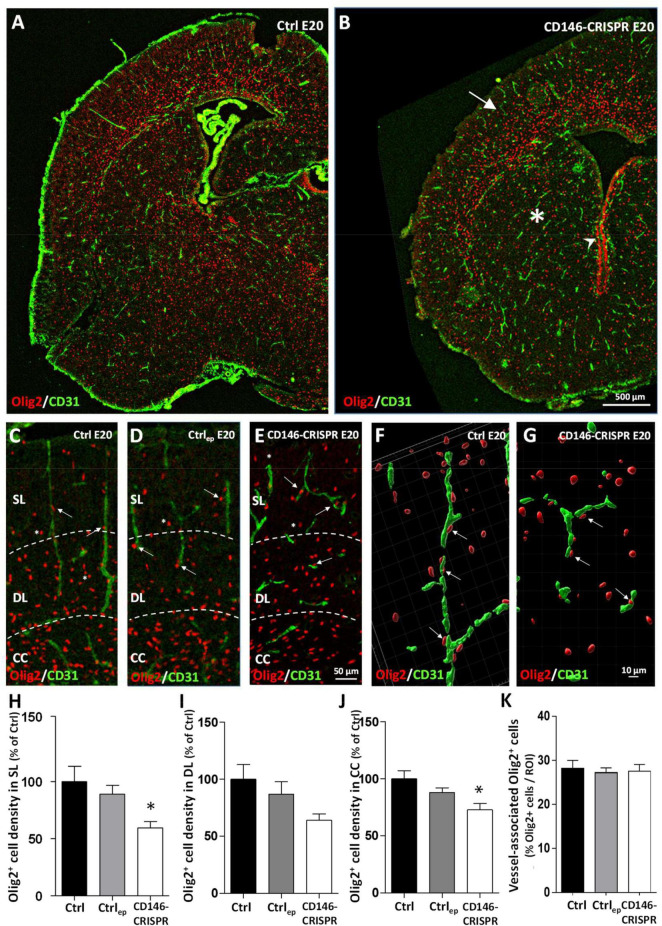
Impact of placental repression of CD146 on the cortical positioning of Olig2^+^ cells in E20 fetuses. Visualization at low magnification of microvessels (CD31 immunolabeling) and oligodendrocytes (Olig2 immunolabeling) in the developing brain of control (no electroporation; Ctrl; **A**) and electroporated CD146-CRISPR (CD146-CRISPR; **B**) fetuses. Note the low density of Olig2^+^ cells in the developing cortex (arrow) and the striatum (asterisk) of CD146-CRISPR fetuses. Visualization at high magnification of microvessels (CD31 immunolabeling) and oligodendrocytes (Olig2 immunolabeling) in the developing cortex of Ctrl **(C)**, electroporated control (control GFP plasmids; Ctrl_ep_; **D**), and CD146-CRISPR **(E)** fetuses at E20. Note the loss of the preferential radial organization of cortical microvessels in CD146-CRISPR fetuses and the proximity of several Olig2^+^ cells with cortical microvessels in all groups (arrows). Three-dimensional map reconstruction from Z-stack acquisitions of Olig2^+^ cells and a cortical microvessels from Ctrl **(F)** and CD146-CRISPR **(G)** fetuses. Quantification of the density of Olig2^+^ cells in the superficial cortical layers (SL; **H**), the deep cortical layers (DL; **I**) and the corpus callosum (CC; **J**) of the developing brain from Ctrl, Ctrl_ep_, and CD146-CRISPR fetuses. **(K)** Quantification of the percentage of vessel-associated Olig2^+^ cells in the developing cortex of Ctrl, Ctrl_ep_, and CD146-CRISPR fetuses. Each value represents the mean (± SEM). *p < 0.05 vs. Ctrl. Statistical details are provided in Supplementary Table 3.

Because recent studies showed that (i) oligodendrocytes use radial microvessels to enter the developing cortex (Brosolo et al., 2022) and that (ii) in FAS human fetuses, alcohol impairs the density and the cortical positioning of cortical oligodendrocytes (Marguet et al., 2022), we investigated whether placental repression of CD146 impaired the cortical layering of oligodendrocytes (Figure 7). Double immunohistochemistry experiments targeting Olig2 and CD31 revealed, at low magnification, three main observations (Figures 7A, B). First, in the CD146-CRISPR group, the loss of the radial vascular organization in the developing cortex is associated with a low density of cortical Olig2-positive (Olig2^+^) cells (Figures 7A, B; arrow). Second, a low density of Olig2^+^ cells is also observed in other brain regions such as the developing striatum (Figures 7A, B; asterisk). Third, contrasting with mice from the Ctrl group, numerous Olig2^+^ cells are persisting at the periventricular region of CD146-CRISPR mice (Figures 7A, B; arrowhead). A quantitative morphometric analysis was done to investigate the impact of placental CD146 repression on oligo-vascular interactions (Figures 7C–K). At high magnification, several Olig2^+^ cells invading the developing cortex are in close interaction with radial microvessels in both Ctrl and Ctrl_ep_ mice (Figures 7C, D; arrows). In CD146-CRISPR mice, although cortical microvessels are disorganized, several Olig2^+^ cells are observed associated to microvessels (Figure 7E; arrows). Three-dimensional maps were built to quantify the proportion of vessel-associated Olig2^+^ cells in the different groups (Figures 7F, G). One-way ANOVA analysis showed a significant treatment interaction of CD146-CRISPR electroporation on the density of Olig2^+^ cells in the SL (*F* 5.01; *p = 0.0216) and in the developing CC (*F* 5.425; *p = 0.0169). The density of Olig2^+^ cells was significantly reduced in CD146-CRISPR fetuses (Figures 7H, J). No significant decrease was quantified in the DL (*F* 3.057; *p* = 0.077; Figure 7I). No effect was found regarding the percentage of vessel-associated Olig2^+^ cells (Figure 7K).

### Correlation analysis between cortical vessel density and cortical oligodendrocyte density in mice repressed for placental CD146

**FIGURE 8:**
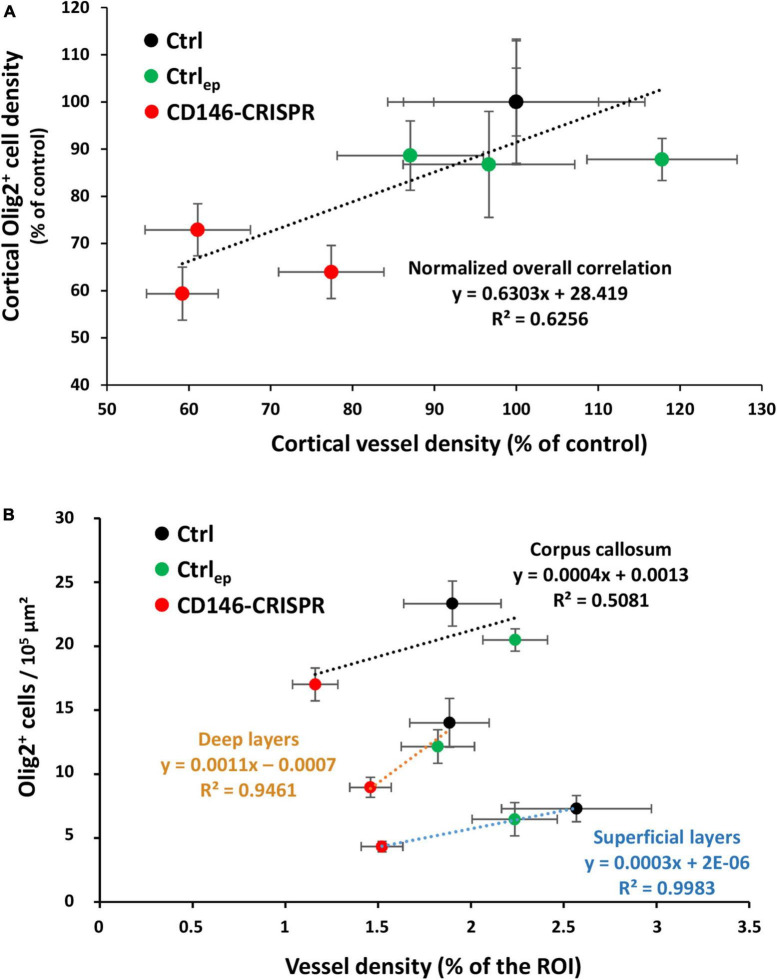
Correlation analysis between cortical vessel density and cortical Olig2^+^ cell density in E20 fetuses from control (no electroporation; Ctrl), electroporated control (control GFP plasmids; Ctrl_ep_), and electroporated CD146-CRISPR (CD146-CRISPR) groups. **(A)** Whole cortical correlation analysis. **(B)** Correlation analysis in the superficial cortical layers (SL), deep cortical layers (DL), and in the corpus callosum (CC). Black, green, and red circles indicate Ctrl, Ctrl_ep_, and CD146-CRISPR groups, respectively. Statistical details are provided in Supplementary Table 3.

Considering that (i) the migration of oligodendrocytes is vessel-associated (Tsai et al., 2016), and (ii) placental repression of CD146 impairs the cortical vasculature (Figures 6F, H) and the cortical density of Olig2^+^ cells (Figures 7H–J), we performed correlation analyses to determine if these different parameters were linked (Figure 8). First, a correlation analysis considering all layers of the sensorimotor cortex showed that the lower the vessel density, the lower the Olig2^+^ cell density (*p* < 0.05; *R*^2^ = 0.6256; Figure 8A). In particular, animals from the CD146-CRISPR group (red points) are clustered in the lowest part of the correlation line when compared to Ctrl (black points) and Ctrl_ep_ (green points) groups (Figure 8A). Second, a correlation analysis considering separately the SL, DL, and CC regions showed that, contrasting with CC (*R*^2^ = 0.5081; Figure 8B), a marked positive correlation was significantly found in SL (*p* < 0.05; *R*^2^ = 0.9983; Figure 8B).

## Discussion

### Expression of CD146 in human and mouse placenta and physiological roles

**FIGURE 9:**
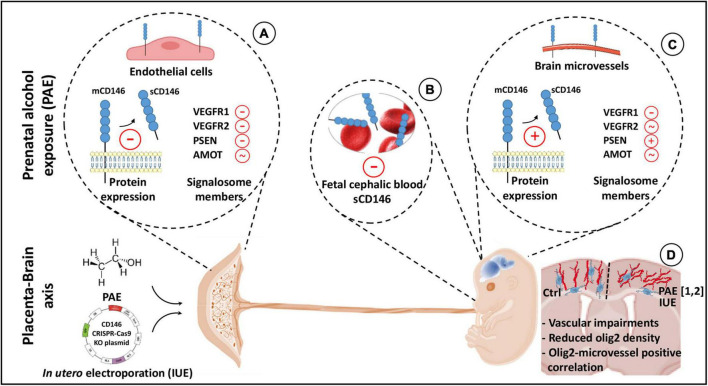
Graphical abstract summarizing highlights of the study. In humans and mice, the proangiogenic factor CD146 is expressed by the placenta. Both membrane and soluble forms are detected. Prenatal alcohol exposure (PAE) reduces soluble CD146 and impairs the expression of different protein members of its signalosome **(A)**. Circulating soluble CD146 is detected in the cephalic blood at embryonic day 20 (E20) and 2 days after birth (P2). Levels are decreased by PAE **(B)**. In fetal (E20) and postnatal (P2) brains, CD146 expression is associated with microvessels. At E20, PAE increases soluble CD146 in the cortex and, when compared to placenta, differently impairs its signalosome **(C)**. A targeted repression of placental CD146 mimics several cortical neurodevelopmental defects induced by PAE (1: Lecuyer et al., 2017; 2: Brosolo et al., 2022), i.e., vascular disorganization and oligodendrocyte mispositioning. Vascular and oligodendrocyte defects are correlated **(D)**.

Previous studies performed in mice and humans reported the effects of CD146 on placental physiology. In mice, intrauterine injections of monoclonal CD146 antibodies caused pregnancy failure (Liu et al., 2008), while in humans soluble CD146 inhibits trophoblast migration (Kaspi et al., 2013). Consistent with these studies, recent data have shown that endothelial cells derived from human placenta express CD146 and that it contributes to vasculogenesis and angiogenesis (Rossi et al., 2019; Gao et al., 2020). In the present study, qRT-PCR experiments revealed that in human villous extracts, CD146 mRNAs are clearly detected at different gestational stages. As found for PlGF, used as an internal control, CD146 mRNA expression increased between first trimester and term placenta; this result was also found for the protein. Such increase could be related to the exponential vascular development occurring in the placenta from the second trimester (Burton et al., 2009). However, the Western blot experiments clearly revealed that only the membrane forms of CD146 increased with gestational age. Indeed, the expression of the soluble form, which is generated by proteolytic cleavage (Stalin et al., 2016), remained stable. The same experiments conducted in mouse placenta showed a similar expression pattern of both membrane and soluble forms of CD146. In addition, the immunohistochemistry experiments indicated that in mouse placenta CD146 is expressed by endothelial cells from the labyrinth zone (Figure 9). Altogether, these data indicate that along gestational stages the expression patterns of membrane and soluble forms of CD146 are different, supporting a regulated processing of CD146. Consistent with this hypothesis, soluble CD146 comprises the overall extracellular portion of the membrane CD146 that is cleaved by matrix metalloproteinases (Boneberg et al., 2009; Leroyer et al., 2019). At a functional level, the fact that CD146 (i) exists in a soluble form, (ii) exerts angiogenic activities (Kebir et al., 2010), and (iii) is a coreceptor of VEGF-R2 (Wang et al., 2020) prompted us to determine whether soluble CD146 can be detected in fetal blood, as previously shown for circulating PlGF originating from the placenta (Lecuyer et al., 2017).

### Expression patterns of CD146 in fetal blood and brain

To reinforce the hypothesis of a contribution of soluble CD146 in a proangiogenic placenta–brain axis, we first investigated whether sCD146 could be detected by ELISA in fetal blood. Indeed, while the literature has shown that sCD146 levels are detectable in pregnant women and significantly higher in women presenting a history of recurrent fetal losses (Kaspi et al., 2013), we did not find evidence in the literature in favor of circulating soluble CD146 in fetuses. ELISA experiments performed in mice at E20 revealed sCD146 levels in the blood of fetuses. Interestingly, blood levels of soluble CD146 decreased after birth, supporting a maternal and/or placental contribution in circulating sCD146, as shown for PlGF (Lecuyer et al., 2017). Regarding the developing brain, the expression of CD146 was quantified at several fetal and postnatal stages. qRT-PCR experiments revealed no modifications of CD146 mRNA expression from E15 to P20. In contrast, Western blot experiments showed significant age-dependent differences. Indeed, mCD146 expression was much higher in fetal versus postnatal stages. Several hypothesis could be raised to interpret these data. First, part of the CD146 proteins detected in the fetal brain could have a peripheral origin, this extra-cerebral fraction of CD146 being lost after delivery. Consistent with this hypothesis, it has been shown in the literature the existence of circulating CD146-positive cells such as endothelial progenitor cells (Delorme et al., 2005). Second, the post-translational regulation of CD146 expression could change in the brain between fetal and postnatal stages. Even if the post-translational regulation of CD146 in the fetal brain is clearly far to be understood, this hypothesis could also be plausible since it has been shown in cancer cells that post-translational modifications of CD146 (glycosylation) extends significantly its half-life (Sumardika et al., 2018). Contrasting with the placenta, the soluble form of CD146 was poorly detected in the brain at both fetal and postnatal stages. The immunohistochemistry experiments showed that CD146 distribution was restricted to brain microvessels (Figure 9). Interestingly, immunolabeling was significantly reduced when animals were previously perfused with PBS. Considering ELISA results that showed a decrease of CD146 levels in the cephalic fetal blood after parturition, these data support that part of the vascular CD146 immunoreactivity would be circulating CD146 and would have a placental origin. Consistent with this hypothesis, a recent study has shown that circulating levels of soluble CD146 are altered in women with placenta-mediated pregnancy complications (Bouvier et al., 2022). Moreover, in a mouse model of preeclampsia, it has also been shown that lower circulating VEGF/PlGF protein levels (two proteins belonging to the CD146 signalosome) are associated to reduced brain cortex angiogenesis (Troncoso et al., 2023). Such a distribution pattern of CD146 looks very similar to the PlGF pattern: (i) CD146 and PlGF (Cao et al., 1997) are strongly expressed in the placenta, and (ii) circulating CD146 and PlGF (Dabrowski et al., 2019) are detected in the blood of fetuses. As recent studies have shown that PlGF is involved in the control of fetal brain angiogenesis (Lecuyer et al., 2017), that CD146 interacts with VEGF-R2 (Wang et al., 2020), that placental PlGF expression is impaired by *in utero* alcohol exposure (Lecuyer et al., 2017), and that PAE impairs cortical angiogenesis in human neonates (Jégou et al., 2012), we investigated the effect of PAE on CD146 expression.

### PAE impairs CD146 expression in the placenta, fetal blood, and developing brain

In the placenta, alcohol did not modify the expression of CD146 mRNA or the membrane form of the protein. In contrast, soluble CD146 expression was markedly decreased (Figure 9). Because soluble CD146 results from the proteolytic cleavage of mCD146 by metalloproteinases (Boneberg et al., 2009), these data suggest that alcohol would alter the CD146 cleavage. Consistent with this hypothesis, a transcriptomic study from Savage’s group showed that, using a voluntary drinking paradigm, moderate alcohol consumption by pregnant rats inhibited the expression of two metalloproteinases in the placenta (Rosenberg et al., 2010). Moreover, the present data showed that PSEN-1, a protease involved in the proteolytic processing of CD146 (Stalin et al., 2016), was also inhibited by PAE. Similarly, ELISA experiments showed that circulating levels of soluble CD146 were reduced in the blood of *in utero* alcohol-exposed neonates (Figure 9), reinforcing the hypothesis of a contribution of the placenta to circulating levels of soluble CD146 in the fetal blood. In contrast, in the fetal brain, while the expression of CD146 mRNA was not impacted by PAE, the expression of the soluble form of CD146 was significantly increased (Figure 9). Such opposite effects of alcohol between the placenta and fetal brain would reflect a compensatory process occurring in the fetal brain. Consistent with this hypothesis, a regression analysis showed that the sCD146/mCD146 ratio in the placenta is decreased in a same range than sCD146 levels in the cephalic blood whereas slopes values of sCD146/mCD146 ratios between placenta and fetal brain are opposite. Such compensatory mechanisms have been already described in the literature. For example, at a functional level, a compensatory mechanism has been described between pulsatility of the middle cerebral artery of the fetus and the umbilical artery in a context of *in utero* growth retardation (IUGR; Monteith et al., 2017). Authors suggested that the resulting cerebroplacental vascular dysfunction would have a diagnosis value in differentiating fetuses at-risk of IUGR (Monteith et al., 2017). In agreement with this hypothesis of functional link between placenta and brain, the present data revealed that PAE also impaired the expression of several proteins from the VEGFR1/R2 signalosome. In particular, while PSEN-1 expression was decreased in the placenta, it was markedly increased in the fetal brain. Because the expression of soluble CD146 results from sequential extracellular and intramembrane cleavages mediated by matrix metalloproteinase and presenilin-1, respectively (Stalin et al., 2016), it would be interesting to investigate the effects of PAE on metalloproteinase and PSEN-1 activities in the placenta and fetal brain.

### Placental repression of CD146 alters the microvasculature of the fetal brain

Functional interactions between the placenta and fetal brain have been recently evidenced regarding the proangiogenic family PlGF/VEGF (Lecuyer et al., 2017). In mice, targeted placental repression of PlGF resulted in a marked decrease in VEGF-R1 expression in the fetal brain and disorganization of microvessels in the developing cortex (Lecuyer et al., 2017). These results were confirmed by a study from Luna and coworkers who showed that *pgf* knockout mice also presented disorganized cortical microvessels (Luna et al., 2016). In humans, a strong correlation was found between angiogenesis defects characterized in the placenta of alcohol-consuming pregnant women and vascular development in the cortex of fetuses (Lecuyer et al., 2017): the more the placental vascularization is reduced, the more the fetal cortical vasculature is disorganized. The fact that (i) CD146 is strongly expressed by the placenta, (ii) soluble CD146 is detected in fetal blood, (iii) soluble CD146 is a coreceptor of VEGF-R2 in tumoral models (Jiang et al., 2012; Stalin et al., 2016), (iv) PAE disrupted the expression of soluble CD146 and several members of its signalosome, and (v) PAE impaired an angiogenic “placenta–brain” axis (Sautreuil et al., 2019) suggested a functional contribution of placental sCD146 in fetal brain angiogenesis. To test this hypothesis, we performed targeted repression of CD146 in the placenta using IUE of CD146 CRISPR/Cas9 constructs. Data revealed a marked alteration of the fetal brain vasculature (Figure 9). In particular, cortical microvessels were disorganized (loss of the radial orientation), and their density was significantly reduced. These results mimicked some vascular impairments described in FAS infants (Jégou et al., 2012; Supplementary Figure 3), and they constitute the first evidence supporting that placental CD146 is involved in the control of fetal brain angiogenesis. In addition, they are consistent with data from the literature, which showed that targeting soluble CD146 with a neutralizing antibody inhibits vascularization, growth and survival of CD146-positive tumors (Stalin et al., 2016), while disruption of CD146 impairs pulmonary vascular remodeling in chronic hypoxic mice (Luo et al., 2019).

### When altered angiogenesis reflects neurodevelopmental impairments

In the last decade, it has been demonstrated in both human fetuses and preclinical models that *in utero* alcohol exposure impairs angiogenesis and disorganizes cortical microvessels (Jégou et al., 2012). Moreover, it is now established that brain microvessels constitute guidance supports for migrating nervous cells, i.e., interneurons (Won et al., 2013; Léger et al., 2020b) and oligodendrocytes (Tsai et al., 2016). Consequently, alcohol-induced microvascular impairments would contribute, almost in part, to the alcohol-induced neurodevelopmental defects. Consistent with this hypothesis, literature recently showed that PAE modifies endothelial protease activity in cortical microvessels, impairs the vessel-associated migration of GABAergic interneurons and the positioning and differentiation of oligodendrocytes in both human fetuses (Marguet et al., 2020, 2022) and in a murine FASD model (Léger et al., 2020a; Darbinian et al., 2021; Brosolo et al., 2022). In agreement with these data, there are more and more evidence supporting that during brain development the vessel-associated migration of oligodendrocyte precursors is closely related to their differentiation process (Xia and Fancy, 2021). In the present study, data showed that, in addition to the vascular disorganization, the placental repression of CD146 resulted in a marked decrease of the density of Olig2^+^ cells in SL (Figure 9). Interestingly, despite marked modifications on cell density and positioning, 3D maps showed no significant effect regarding the percentage of Olig2^+^ cells associated to microvessels suggesting that PAE alcohol would more impact the migration route rather than mechanisms leading to oligo-vascular association. Correlation analysis performed in the whole cortical thickness or in the CC, DL, and SL provided several indications. First, the more the vessel density is reduced, the more the oligodendrocyte density is reduced, reinforcing the hypothesis of a functional link between these two observations also observed in PAE fetuses (Lecuyer et al., 2017; Brosolo et al., 2022). Second, *R*^2^ coefficients from correlation analyses are particularly high in SL, where the vascular disorganization is obvious. Third, all fetuses from CD146-repressed placentas are clustered in the same area of the correlation line corresponding to the lowest densities of oligodendrocytes and microvessels. Altogether, these data constitute the first demonstration that the pro-angiogenic placental factor CD146 would contribute to neurodevelopmental impairments.

### CD146, a placental biomarker candidate for FASD diagnosis?

Despite active primary prevention in several countries, alcohol consumption during pregnancy is a leading cause of lifelong developmental and physical disabilities and behavioral problems (Popova et al., 2017). This is in part explained by the economic emergence of several countries, increased spending power, and facilitated access to alcohol for recreational use and as a vector of social integration (World Health Organization [WHO], 2018). In Europe, one in four women consume alcohol during pregnancy (World Health Organization [WHO], 2018). FAS is the most severe manifestation of FASD, and it is associated with several clinical signs, such as craniofacial dysmorphism, growth retardation and neurodevelopmental deficits (Joya et al., 2015). Based on these criteria, a perinatal diagnosis of FAS is possible. However, this is not the case for most FASD children who are frequently misdiagnosed until they are school age (Chasnoff et al., 2015). Indeed, although most of them do not exhibit the characteristic physical features of FAS, they are not devoid of neurobehavioral disabilities. Thus, an early diagnosis of FASD infants, who are estimated to be between 5- and 10-fold more numerous than FAS children (World Health Organization [WHO], 2018), is a challenging objective for clinicians to avoid the loss of precious years of care (Chasnoff et al., 2015).

Neuroplacentology constitutes an emerging field of research investigating the role of placenta in brain development (Freedman et al., 2022; Gardella et al., 2022). In particular, it has been shown that placenta contributes to the control of brain angiogenesis (Lecuyer et al., 2017) and that PAE alters the inter-organ “placenta–brain” transcriptomic signature (Sautreuil et al., 2023). Concurrently, several research groups have shown that a proper brain angiogenesis is a prerequisite for a correct neurodevelopment (Won et al., 2013; Tsai et al., 2016). Moreover, in recently patented data, it has been shown that the placenta, by releasing proangiogenic factors, could constitute a promising source of circulating biomarkers for the early diagnosis of FASD (WO2016207253, Gonzalez et al., 2016 and WO2018100143, Gonzalez et al., 2018). Several recent studies have shown that CD146 has a prognostic biomarker value for the diagnosis of cancer (Lei et al., 2015; Rapanotti et al., 2021; Du et al., 2022). This biomarker potential can be related to the angiogenic properties of CD146 (Joshkon et al., 2020), a physiological function that is dysregulated by PAE (Lecuyer et al., 2017; Léger et al., 2020a). Consequently, in addition to provide new evidence in favor of a physiological role of placental CD146 in the fetal brain angiogenesis, the present study also suggests that soluble CD146 could have a biomarker value for the detection of alcohol-induced dysfunction of the “placenta/brain” axis. However, because neurodevelopmental abnormalities are not synonymous of neurodevelopmental disorders, next research avenues would be to perform a postnatal study comparing behavioral troubles induced by PAE with those of pups from CD146-repressed placentas.

Regarding sex differences, previous pre-clinical studies reported that the alcohol-induced neurovascular alterations impacting GABAergic interneurons (Léger et al., 2020a) or oligodendrocytes (Brosolo et al., 2022) were not significantly different between males and females. These sex-independent results regarding molecular data have also been observed in human neuroanatomical studies (Fraize et al., 2023; Pfefferbaum et al., 2023). However, they also clearly differ from clinical studies that reported sex-differences regarding alcohol-induced neurodevelopmental disorders (Flannigan et al., 2023). Altogether, these studies suggest that while molecular and/or anatomical defects may be similar in both sexes, their expression in term of behavioral troubles could be different. Interestingly, the placenta could contribute to these sex-related behavioral troubles (Bale, 2016).They also reinforce that molecular and mechanistic pre-clinical studies should have to be continued with behavioral studies focusing in neurodevelopmental disorders.

## Conclusion

In conclusion, the present study shows that during human gestation, the placenta expresses the membrane and soluble forms of the proangiogenic factor CD146. In mice, the expression pattern of CD146 in the placenta is very similar to that described in humans, and the soluble form is also detected in the fetal cephalic blood. *In utero* alcohol exposure dysregulates the expression of the soluble form of CD146 as well as of other members of the VEGF-R1/R2 signalosome in both placenta and fetal brain. Moreover, a targeted repression of CD146 in placenta results in impairments of vascular development in the fetal cortex and of the positioning of oligodendrocytes whose migration is vessel-associated. Considering that it has been recently shown that PAE alters both fetal brain angiogenesis and the oligodendrocyte lineage in mouse FASD models and in FAS human fetuses, the present study reinforce the emerging notion of a functional “placenta–brain” axis impacting neurovascular development. They also support that the soluble form of CD146 could constitute a placental angiogenic factor with a biomarker value for the early diagnosis of alcohol-induced fetal brain disorders.

## Data availability statement

The original contributions presented in this study are included in this article/Supplementary material, further inquiries can be directed to the corresponding author.

## Ethics statement

Placenta were obtained from Port-Royal Maternity, the Mutualist Institute Montsouris, the Private Hospital of Antony, the Antoine-Béclère Hospital, and the Beaujon Hospital after obtaining written consent from informed patients and approval from our local ethics committee (CPP: 2015-May-13909). The studies were conducted in accordance with the local legislation and institutional requirements. The participants provided their written informed consent to participate in this study. The animal study was approved by authorization no. APAFIS#22136-2019092013438607 v4 from the Ministère de l’Enseignement Supérieur, de la Recherche et de l’Innovation. The study was conducted in accordance with the local legislation and institutional requirements.

## Author contributions

CS: Data curation, Formal analysis, Investigation, Methodology, Writing – original draft, Writing – review & editing. MLo: Conceptualization, Data curation, Formal analysis, Investigation, Validation, Writing – original draft, Writing – review & editing. JD: Investigation, Methodology, Validation, Writing – original draft, Writing – review & editing. ALe: Investigation, Methodology, Writing – review & editing. MLy: Methodology, Writing – review & editing. FJ: Formal analysis, Investigation, Methodology, Writing – original draft. MLl: Investigation, Writing – review & editing. SB: Visualization, Writing – review & editing. SM: Conceptualization, Validation, Visualization, Writing – review & editing. ALa: Validation, Visualization, Writing – original draft, Writing – review & editing. CB-L: Investigation, Methodology, Visualization, Writing – review & editing. SG: Data curation, Visualization, Writing – original draft, Writing – review & editing. BG: Conceptualization, Data curation, Formal analysis, Funding acquisition, Project administration, Resources, Supervision, Validation, Writing – original draft, Writing – review & editing.

## References

[B1] Alsat E. Guibourdenche J. Couturier A. Evain-Brion D. (1998). Physiological role of human placental growth hormone. *Mol. Cell Endocrinol.* 140 121–127. 9722179 10.1016/s0303-7207(98)00040-9

[B2] An Y. Wei N. Cheng X. Li Y. Liu H. Wang J. (2020). MCAM abnormal expression and clinical outcome associations are hightly cancer dependant as revealed through pan-cancer analysis. *Brief. Bioinform.* 21 709–718. 10.1093/bib/bbz019 30815677

[B3] Bale T. (2016). The placenta and neurodevelopment: Sex differences in prenatal vulnerability. *Dialogues Clin. Neurosci.* 18 459–464. 28179817 10.31887/DCNS.2016.18.4/tbale PMC5286731

[B4] Bardin N. Francès V. Combes V. Sampol J. Dignat-George F. (1998). CD146: Biosynthesis and production of a soluble form in human cultured endothelial cells. *FEBS Lett.* 421 12–14. 10.1016/s0014-5793(97)01455-5 9462829

[B5] Boneberg E. Illges H. Legler D. Fürstenberger G. (2009). Soluble CD146 is generated by ectodomain shedding of membrane CD146 in a calcium-induced, matrix metalloprotease-dependant process. *Microvasc. Res.* 78 325–331. 10.1016/j.mvr.2009.06.012 19615385

[B6] Bouvier S. Traboulsi W. Blois S. Demattei C. Joshkon A. Mousty E. (2022). Soluble CD146 is increased in preeclampsia and interacts with galectin-1 to regulate trophoblast migration through VEGFR2 receptor. *F S Sci*. 3 84–94. 10.1016/j.xfss.2021.11.002 35559998

[B7] Brosolo M. Lecointre M. Laquerrière A. Janin F. Genty D. Lebon A. (2022). In utero alcohol exposure impairs vessel-associated positioning and differentiation of oligodendrocytes in the developing neocortex. *Neurobiol. Dis.* 171:105791. 10.1016/j.nbd.2022.105791 35760273

[B8] Burton G. Charnock-Jones D. Jauniaux E. (2009). Regulation of vascular growth and function in the human placenta. *Reproduction* 138 895–902. 19470597 10.1530/REP-09-0092

[B9] Cao Y. Ji W. Qi P. Rosin A. Cao Y. (1997). Placenta growth factor: Identification and characterization of a novel isoform generated by RNA alternative splicing. *Biochem. Bioph. Res. Co* 235 493–498. 10.1006/bbrc.1997.6813 9207183

[B10] Chasnoff I. Wells A. King L. (2015). Misdiagnosis and missed diagnoses in foster and adopted children with prenatal alcohol exposure. *Pediatrics* 135 264–270. 10.1542/peds.2014-2171 25583914

[B11] Dabrowski F. Lipa M. Bartoszewicz Z. Wielgos M. Bomba-Opon D. (2019). Maternal and neonatal serum expression of the vascular growth factors in hyperglycemia in pregnancy. *J. Matern. Fetal Neonatal. Med.* 15 1–6. 10.1080/14767058.2019.1639666 31307255

[B12] Darbinian N. Darbinyan A. Merabova N. Bajwa A. Tatevosian G. Martirosyan D. (2021). Ethanol-mediated alterations in oligodendrocyte differentiation in the developing brain. *Neurobiol. Dis.* 148:105181. 10.1016/j.nbd.2020.105181 33189883 PMC7856167

[B13] Delorme B. Basire A. Gentile C. Sabatier F. Monsonis F. Desouches C. (2005). Presence of endothelial progenitor cells, distinct from mature endothelial cells, within human CD146+ blood cells. *Thromb. Haemost.* 94 1270–1279. 10.1160/TH05-07-0499 16411405

[B14] Du X. Zhang Q. Wang S. Chen X. Wang Y. (2022). MCAM is associated with metastasis and poor prognosis in osteosarcoma by modulating tumor cell migration. *J. Clin. Lab Anal.* 36:e24214. 10.1002/jcla.24214 34961985 PMC8841137

[B15] Flannigan K. Poole N. Cook J. Unsworth K. (2023). Sex-related differences among individuals assessed for fetal alcohol spectrum disorder in Canada. *Alcohol. Clin. Exp. Res.* 47 613–623. 10.1111/acer.15017 36932990

[B16] Fraize J. Fischer C. Elmaleh-Bergès M. Kerdreux E. Beggiato A. Ntorkou A. (2023). Enhancing fetal alcohol spectrum disorders diagnosis with a classifier based on the intracerebellar gradient of volumetric undersizing. *Hum. Brain Mapp.* 44 4321–4336. 10.1002/hbm.26348 37209313 PMC10318202

[B17] Freedman A. Eaves L. Rager J. Gavino-Lopez N. Smeester L. Bangma J. (2022). The placenta epigenome-brain axis: Placental epigenomic and transcriptomic responses that preprogram cognitive impairment. *Epigenomics* 14 897–911. 10.2217/epi-2022-0061 36073148 PMC9475498

[B18] Gao K. He S. Kumar P. Farmer D. Zhou J. Wang A. (2020). Clonal isolation of endothelial colony-forming cells from early gestation chorionic villi of human placenta for fetal tissue regeneration. *World J. Stem Cells* 12 123–138. 10.4252/wjsc.v12.i2.123 32184937 PMC7062038

[B19] Gardella B. Dominoni M. Scatigno A. Cesari S. Fiandrino G. Orcesi S. (2022). What is known about neuroplacentology in fetal growth restriction and in preterm infants: A narrative review of literature. *Front. Endocrinol.* 13:936171. 10.3389/fendo.2022.936171 36060976 PMC9437342

[B20] Gonzalez B. Marret S. Lecuyer M. Laquerrière A. Bekri S. Lesueur C. (2016). *Method for the diagnosis of disorders caused by fetal alcohol syndromes.* Available online at: https://patentscope.wipo.int/search/fr/detail.jsf?docId=WO2016207253

[B21] Gonzalez B. Marret S. Lecuyer M. Laquerrière A. Bekri S. Lesueur C. (2018). *Placental growth factor for the treatment of fetal alcohol syndrome disorders (FASD).* Available online at: https://patentscope.wipo.int/search/en/detail.jsf?docId=WO2018100143

[B22] Haghighi Poodeh S. Salonurmi T. Nagy I. Koivunen P. Vuoristo J. Räsänen J. (2012). Alcohol-induced premature permeability in mouse placenta-yolk sac barriers in vivo. *Placenta* 33 866–873. 10.1016/j.placenta.2012.07.008 22884851

[B23] Jégou S. El Ghazi F. Kwetieu de Lendeu P. Marret S. Laudenbach V. Uguen A. (2012). Prenatal alcohol exposure affects vasculature development in the neonatal brain. *Ann. Neurol.* 72 952–960. 23280843 10.1002/ana.23699

[B24] Jiang T. Zhuang J. Duan H. Luo Y. Zeng Q. Fan K. (2012). CD146 is a coreceptor for VEGFR-2 in tumor angiogenesis. *Blood* 120 2330–2339. 10.1182/blood-2012-01-406108 22718841

[B25] Joshkon A. Heim X. Dubrou C. Bachelier R. Traboulsi W. Stalin J. (2020). Role of CD146 (MCAM) in physiological and pathological angiogenesis-contribution of new antibodies for therapy. *Biomedicines* 8 633–652. 10.3390/biomedicines8120633 33352759 PMC7767164

[B26] Joya X. Garcia-Algar O. Salat-Batlle J. Pujades C. Vall O. (2015). Advances in the development of novel antioxidant therapies as an approach for fetal alcohol syndrome prevention. *Birth Defects Res. A Clin. Mol. Teratol.* 103 163–177. 10.1002/bdra.23290 25131946

[B27] Kaspi E. Guillet B. Percecchi-Marti M. Alfaidy N. Bretelle F. Bertraud-Foucault A. (2013). Identification of soluble CD146 as a regulator of trophoblast migration: Potential role in placental vascular development. *Angiogenesis* 16 329–342. 23108590 10.1007/s10456-012-9317-6

[B28] Kebir A. Harhouri K. Guillet B. Liu J. Foucault-Bertraud A. Lamy E. (2010). CD146 short isoform increases the proangiogenic potential of endothelial progenitor cells in vitro and in vivo. *Circ. Res.* 107 66–75. 10.1161/CIRCRESAHA.109.213827 20448216

[B29] Lecuyer M. Laquerrière A. Bekri S. Lesueur C. Ramdani Y. Jégou S. (2017). PLGF, a placental marker of fetal brain defects after in utero alcohol exposure. *Acta Neuropathol. Commun.* 5 44–64. 10.1186/s40478-017-0444-6 28587682 PMC5461764

[B30] Léger C. Dupré N. Laquerrière A. Lecointre M. Dumanoir M. Janin F. (2020a). In utero alcohol exposure exacerbates endothelial protease activity from pial microvessels and impairs GABA interneuron positioning. *Neurobiol. Dis.* 145:105074. 10.1016/j.nbd.2020.105074 32890773

[B31] Léger C. Dupré N. Aligny C. Bénard M. Lebon A. Henry V. (2020b). Glutamate controls vessel-associated migration of GABA interneurons from the pial migratory route via NMDA receptors and endothelial protease activation. *Cell Mol. Life Sci.* 77 1959–1986. 10.1007/s00018-019-03248-5 31392351 PMC7229000

[B32] Lehmann J. Riethmüller G. Johnson J. (1989). MUC18, a marker of tumor progression in human melanoma, shows sequence similarity to the neural cell adhesion molecules of the immunoglobulin superfamily. *Proc. Natl. Acad. Sci. U.S.A.* 86 9891–9895. 10.1073/pnas.86.24.9891 2602381 PMC298608

[B33] Lei X. Guan C. Song Y. Wang H. (2015). The multifaceted role of CD146/MCAM in the promotion of melanoma progression. *Cancer Cell Int.* 15 3–14. 10.1186/s12935-014-0147-z 25685061 PMC4326486

[B34] Leroyer A. Blin M. Bachelier R. Bardin N. Blot-Chabaud M. Dignat-George F. (2019). CD146 (Cluster of Differentiation 146). *Arterioscler Thromb. Vasc. Bio* 39 1026–1033. 31070478 10.1161/ATVBAHA.119.312653

[B35] Liu Q. Zhang B. Zhao X. Zhang Y. Liu Y. Yan X. (2008). Blockade of adhesion molecule CD146 causes pregnancy failure in mice. *J. Cell Physiol.* 215 621–626. 10.1002/jcp.21341 18288634

[B36] Lui S. Jones R. Robinson N. Greenwood S. Aplin J. Tower C. (2014). Detrimental effects of ethanol and its metabolite acetaldehyde, on first trimester human placental cell turnover and function. *PLoS One* 9:e87328. 10.1371/journal.pone.0087328 24503565 PMC3913587

[B37] Luna R. Kay V. Rtsep M. Khalaj K. Bidarimath M. Peterson N. (2016). Placental growth factor deficiency is associated with impaired cerebral vascular development in mice. *Mol. Hum. Reprod.* 22 130–142. 26646502 10.1093/molehr/gav069 PMC4733225

[B38] Luo Y. Teng X. Zhang L. Chen J. Liu Z. Chen X. (2019). CD146-HIF-1α hypoxic reprogramming drives vascular remodeling and pulmonary arterial hypertension. *Nat. Commun.* 10 3551–3568. 31391533 10.1038/s41467-019-11500-6 PMC6686016

[B39] Marguet F. Brosolo M. Friocourt G. Sauvestre F. Marcorelles P. Lesueur C. (2022). Oligodendrocyte lineage is severely affected in human alcohol-exposed foetuses. *Acta Neuropathol. Commun.* 10 74–88. 35568959 10.1186/s40478-022-01378-9 PMC9107108

[B40] Marguet F. Friocourt G. Brosolo M. Sauvestre F. Marcorelles P. Lesueur C. (2020). Prenatal alcohol exposure is a leading cause of interneuronopathy in humans. *Acta Neuropathol. Commun.* 8 208–226. 10.1186/s40478-020-01089-z 33256853 PMC7706035

[B41] Monteith C. Flood K. Mullers S. Unterscheider J. Breathnach F. Daly S. (2017). Evaluation of normalization of cerebro-placental ratio as a potential predictor for adverse outcome in SGA fetuses. *Am. J. Obstet Gynecol.* 216 285.e1–285.e6. 10.1016/j.ajog.2016.11.1008 27840142

[B42] Pfefferbaum A. Sullivan E. Pohl K. Bischoff-Grethe A. Stoner S. Moore E. (2023). Brain volume in fetal alcohol spectrum disorders over a 20-year span. *JAMA Netw. Open* 6:e2343618. 10.1001/jamanetworkopen.2023.43618 37976065 PMC10656646

[B43] Popova S. Lange S. Probst C. Gmel G. Rehm J. (2017). Estimation of national, regional, and global prevalence of alcohol use during pregnancy and fetal alcohol syndrome: A systematic review and meta-analysis. *Lancet Glob. Health* 5 e290–e299. 10.1016/S2214-109X(17)30021-9 28089487

[B44] Rapanotti M. Cugini E. Nuccetelli M. Terrinoni A. Di Raimondo C. Lombardo P. (2021). MCAM/MUC18/CD146 as a multifaceted warning marker of melanoma progression in liquid biopsy. *Int. J. Mol. Sci.* 22 12416–12438. 10.3390/ijms222212416 34830300 PMC8623757

[B45] Riley E. Infante M. Warren K. (2011). Fetal alcohol spectrum disorders: An overview. *Neuropsychol. Rev.* 21 73–80. 21499711 10.1007/s11065-011-9166-x PMC3779274

[B46] Riley E. Mattson S. Li T. Jacobson S. Coles C. Kodituwakku P. (2003). Neurobehavioral consequences of prenatal alcohol exposure: An international perspective. *Alcohol. Clin. Exp. Res.* 27 362–373. 12605086 10.1097/01.ALC.0000052703.38558.B2

[B47] Rosenberg M. Wolff C. El-Emawy A. Staples M. Perrone-Bizzozero N. Savage D. (2010). Effects of moderate drinking during pregnancy on placental gene expression. *Alcohol* 44 673–690. 20053520 10.1016/j.alcohol.2009.10.002 PMC3654802

[B48] Rossi E. Poirault-Chassac S. Bieche I. Chocron R. Schnitzler A. Lokajczyk A. (2019). Human endothelial colony forming cells express intracellular CD133 that modulates their vasculogenic properties. *Stem Cell Rev. Rep.* 15 590–600. 10.1007/s12015-019-09881-8 30879244

[B49] Sautreuil C. Laquerrière A. Lecuyer M. Brasse-Lagnel C. Jégou S. Bekri S. (2019). Fetal alcohol exposure: When placenta would help to the early diagnosis of child brain impairments. *Med. Sci.* 35 859–865. 10.1051/medsci/2019167 31845877

[B50] Sautreuil C. Lecointre M. Derambure C. Brasse-Lagnel C. Leroux P. Laquerrière A. (2023). Prenatal alcohol exposure impairs the placenta-cortex transcriptomic signature: Dysregulation of angiogenic pathways. *Int. J. Mol. Sci.* 24 13484–13511. 10.3390/ijms241713484 37686296 PMC10488081

[B51] Sers C. Riethmüller G. Johnson J. (1994). MUC18, a melanoma-progression associated molecule, and its potential role in tumor vascularization and hematogenous spread. *Cancer Res.* 54 5689–5694. 7923217

[B52] Shih I. (1994). The role of CD146 (Mel-CAM) in biology and pathology. *J. Pathol.* 189 4–11. 10.1002/(SICI)1096-9896(199909)189:1<4::AID-PATH332>3.0.CO;2-P 10451481

[B53] Stalin J. Harhouri K. Hubert L. Garrigue P. Nollet M. Essaadi A. (2016). Soluble CD146 boosts therapeutic effect of endothelial progenitors through proteolytic processing of short CD146 isoform. *Cardiovasc. Res.* 111 240–251. 10.1093/cvr/cvw096 27170199

[B54] Stalin J. Harhouri K. Hubert L. Subrini C. Lafitte D. Lassitzky J. (2013). Soluble melanoma cell adhesion molecule (sMCAM/sCD146) promotes angiogenic effects on endothelial progenitor cells through angiomotin. *J. Biol. Chem.* 288 8991–9000. 10.1074/jbc.M112.446518 23389031 PMC3610971

[B55] Sumardika I. Youyi C. Kondo E. Inoue Y. Ruma I. Murata H. (2018). β-1,3-Galactosyl-*O*-Glycosyl-Glycoprotein β-1,6-*N*-acetylglucosaminyltransferase 3 increases MCAM stability, which enhances S100A8/A9-mediated cancer motility. *Oncol. Res.* 26 431–444. 28923134 10.3727/096504017X15031557924123 PMC7844831

[B56] Taira E. Nagino T. Taniura H. Takaha N. Kim C. Kuo C. (1995). Expression and functional analysis of a novel isoform of gicerin, an immunoglobulin superfamily cell adhesion molecule. *J. Biol. Chem.* 270 28681–28687. 10.1074/jbc.270.48.28681 7499388

[B57] Troncoso F. Sandoval H. Ibañez B. López-Espíndola D. Bustos F. Tapia J. (2023). Reduced Brain Cortex Angiogenesis in the Offspring of the Preeclampsia-Like Syndrome. *Hypertension* 80 2559–2571. 37767691 10.1161/HYPERTENSIONAHA.123.21756 PMC7618073

[B58] Tsai H. Niu J. Munji R. Davalos D. Chang J. Zhang H. (2016). Oligodendrocyte precursors migrate along vasculature in the developing nervous system. *Science* 351 379–384. 26798014 10.1126/science.aad3839 PMC5472053

[B59] Wang Z. Xu Q. Zhang N. Du X. Xu G. Yan X. (2020). CD146, from a melanoma cell adhesion molecule to a signaling receptor. *Signal. Transduct. Target Ther.* 5 148–163. 32782280 10.1038/s41392-020-00259-8 PMC7421905

[B60] Won C. Lin Z. Kumar T. Li S. Ding L. Elkhal A. (2013). Autonomous vascular networks synchronize GABA neuron migration in the embryonic forebrain. *Nat. Commun.* 4 2149–2162. 10.1038/ncomms3149 23857367 PMC3763945

[B61] World Health Organization [WHO] (2018). *Global status report on alcohol and health.* Available online at: https://www.who.int/substance_abuse/publications/global_alcohol_report/gsr_2018/en 2018

[B62] Xia W. Fancy S. (2021). Mechanisms of oligodendrocyte progenitor developmental migration. *Dev. Neurobiol.* 81 985–996. 34643996 10.1002/dneu.22856

