# Supplementary material for: Expression of placental CD146 is dysregulated by prenatal alcohol exposure and contributes in cortical vasculature development and positioning of vessel-associated oligodendrocytes
Source: Front Cell Neurosci. 2024 Jan 10;17:1294746. doi: 10.3389/fncel.2023.1294746 (PMC10806802; doi:10.3389/fncel.2023.1294746)
Supplement: Supplementary file 1 [file Table_1.docx]

**Supplementary Table 1.** Origin and characteristics of the primary antibodies used for the immunohistochemical and Western blot studies performed in mouse tissues.

| Antibodies | Trade reference | Purified species | Supplier | Dilution | Solution of incubation |
| --- | --- | --- | --- | --- | --- |
| Angiomotin/  Amot | Mouse WB: (G-12) sc-515262 | Mouse | Santa Cruz Biotechnology | 1/1000 | Milk (5% in TBST) |
| β-actin | Mouse WB: A5441 | Mouse | Sigma-aldrich | 1/5000 | Milk (5% in TBST) |
| CD146  (Mouse studies) | WB: [P1H12] ab24577 | Mouse | abcam | 1/1000 | Milk (5% in TBST) |
|  | IHC:  PA1736 | Rabbit | Boster Bio | 1/400 | BSA (1% in PBS) |
| CD146  (Human studies) | WB-IHC:  PA1736 | Rabbit | Boster Bio | 1/2000 | Milk (5% in TBST) |
| CK7 | Human IHC: M7018 | Mouse | Agilent Dako | 1/200 | BSA (1% in PBS) |
| GFP | Mouse WB: ab6673 | Goat | abcam | 1/1000 | Milk (5% in TBST) |
| Glut1 | Mouse IHC:  sc-1605 | Goat | Santa Cruz Biotechnology | 1/200 | BSA (1% in PBS) |
| Olig2 | Mouse IHC: ab136253 | Rabbit | abcam | 1/200 | BSA (1% in PBS) |
| CD31 | Mouse IHC:  cat 550274 | Rat | BD-Pharmigen | 1/400 | BSA (1% in PBS) |
| Presenilin-1/  PSEN-1 | Mouse WB: ARP58941 | Rabbit | Aviva systems biology | 1/1000 | Milk (5% in TBST) |
| VEGF-R1 | Mouse WB: (C-17) sc-316 | Rabbit | Santa Cruz Biotechnology | 1/1000 | Milk (5% in TBST) |
| VEGF-R2 | Mouse WB: (N-931) sc-505 | Rabbit | Santa Cruz Biotechnology | 1/1000 | Milk (5% in TBST) |
| Vinculin | Human WB: V9131 | Mouse | Sigma-Aldrich | 1/1000 | Milk (5% in TBST) |
