# Supplementary material for: Expression of placental CD146 is dysregulated by prenatal alcohol exposure and contributes in cortical vasculature development and positioning of vessel-associated oligodendrocytes
Source: Front Cell Neurosci. 2024 Jan 10;17:1294746. doi: 10.3389/fncel.2023.1294746 (PMC10806802; doi:10.3389/fncel.2023.1294746)
Supplement: Supplementary file 2 [file Table_2.docx]

**Supplementary Table 2.** Primer sequences designed for qRT-PCR experiments.

| **Gene name** | **Forward primers** | **Reverse primers** |
| --- | --- | --- |
| ***Mouse*** | | |
| **CD146** | 5’-GGGCCTCAGGCAACTTCA-3’ | 5’-TTGGTGCACACGGAAAATCA-3’ |
| **GAPDH** | 5’-TCATGGCCTTCCGTGTTCCTA-3’ | 5’-CCTGCTTCACCACCTTCTTGA-3’ |
| ***Human*** | | |
| **CD146** | 5’-CAGGGAAGCAGGAGATCACG-3’ | 5’-CAGGAGGCCCATCTCTTCTG-3’ |
| **PGF** | 5’-GCTCGTCAGAGGTGGAAGTGGT-3’ | 5’-CTCGCTGGGGTACTCGGACA-3’ |
| **PSEN-1** | 5’-GGACAACCACCTGAGCAATAC-3’ | 5’-AAGGCTCCGTCTGTCGTTG-3’ |
| **RPL13** | 5’-AAGGTCGTGCGTCTGAAG-3’ | 5’-GAGTCCGTGGGTCTTGAG-3’ |
| **RPLPO** | 5’-AACATCTCCCCCTTCTCCT-3’ | 5’-ACTCGTTTGTACCCGTTGAT-3’ |
| **VEGR-R1** | 5’-TCCCTTATGATGCCAGCAAGT-3’ | 5’-CCAAAAGCCCCTCTTCCAA-3’ |
| **VEGF-R2** | 5’-CACACAGTGGCCATCAGC-3’ | 5’-CGATGACGATGGTGACGTT-3’ |
