# Supplementary material for: Expression of placental CD146 is dysregulated by prenatal alcohol exposure and contributes in cortical vasculature development and positioning of vessel-associated oligodendrocytes
Source: Front Cell Neurosci. 2024 Jan 10;17:1294746. doi: 10.3389/fncel.2023.1294746 (PMC10806802; doi:10.3389/fncel.2023.1294746)
Supplement: Supplementary file 3 [file Table_3.docx]

**Supplementary Table 3.** Statistical analyses.

| **Experiments** | **Test** | **n**  **independents experiments** | **p value**  ***p<0.05; **p<0.01; ***p<0.001; ****p<0.0001**  **ns, not significant** |
| --- | --- | --- | --- |
| Figure 1A  mRNA CD146 | One way ANOVA  Tukey’s multiple comparison test | n=4 | ANOVA F 10.33 p=0.0030, **  Tukey’s test  8 GW vs 13 GW  p=0.8314, ns  8 GW vs 40 GW  p=0.0047, **  13 GW vs 40 GW  p=0.0138, * |
| Figure 1B  mRNA PGF | One way ANOVA  Tukey’s multiple comparison test | n=4 | ANOVA F 70.90 p<0.0001, ****  Tukey’s test  8 GW vs 13 GW  p=0.9446, ns  8 GW vs 40 GW  p<0.0001, ****  13 GW vs 40 GW  p<0.0001, **** |
| Figure 1C  mRNA VEGF-R1 | One way ANOVA  Tukey’s multiple comparison test | n=4 | ANOVA F 10.83 p=0.0040, **  Tukey’s test  8 GW vs 13 GW  p=0.9642, ns  8 GW vs 40 GW  p<0.0063, **  13 GW vs 40 GW  p<0.0092, ** |
| Figure 1D  mRNA VEGF-R2 | One way ANOVA  Tukey’s multiple comparison test | n=4 | ANOVA F 6.382 p=0.0188, *  Tukey’s test  8 GW vs 13 GW  p=0.9627, ns  8 GW vs 40 GW  p=0.0258, *  13 GW vs 40 GW  p=0.0388, * |
| Figure 1E  mRNA PSEN-1 | One way ANOVA  Tukey’s multiple comparison test | n=4 | ANOVA F 67.82 p<0.0001, ****  Tukey’s test  8 GW vs 13 GW  p=0.9902, ns  8 GW vs 40 GW  p<0.0001, ****  13 GW vs 40 GW  p<0.0001, **** |
| Figure 1F  CD146 | Two-way ANOVA  Bonferroni post-test | n=4 | ANOVA  Interaction F 4.366 p=0.0285, *  Gestational weeks F 23.95  p=0.0001, ****  CD146 forms F 5.347  p=0.0150, *  Bonferroni’s  For 8 GW  mCD146 vs sCD146 p=0.4226, ns  For 13 GW  mCD146 vs sCD146 p=0.3194, ns  For 40 GW  mCD146 vs sCD146 p=0.0002, *** |
| Figure 2A  mRNA CD146 | One-way ANOVA  Tukey’s post hoc test | n=5 | ANOVA F 5.269 p=0.0228, *  Tukey’s post hoc test  E15 vs E17 p=0.9117, ns  E15 vs E20 p=0.0580, ns  E17 vs E20 p=0.0280, * |
| Figure 2B  CD146 vs sCD146 | Two-way ANOVA  Bonferroni post-test | n=3 | ANOVA  Interaction F 26.90 p=0.0001, ****  Protein Levels F 53.43  p<0.0001, ****  Age F 11.85  p=0.0040, ***  Bonferroni’s  For E15  CD146 vs sCD146 p>0.05, ns  For E17  CD146 vs sCD146 p<0.001, ***  For E20  CD146 vs sCD146 p<0.001, *** |
| Figure 3A  mRNA CD146 | One way ANOVA  Tukey’s post hoc test | n=3 | ANOVA F 2.373 p=0.0661, ns  Tukey’s  P10 vs P20 p=0.0356, * |
| Figure 3B  CD146 | Unpaired t test | Fetal stages n=17  Postnatal stages n=14 | Fetal *vs* postnatal stages p<0.0001, **** |
| Figure 3G  CD146/CD31 | One-way ANOVA  Tukey’s post hoc test | n=3 | ANOVA F 29.12  p<0.0001, ****  Tukey’s  E20 *vs* npP2 p=0.0092, **  E20 *vs* pP2 p<0.0001, ****  npP2 *vs* pP2 p<0.0001, **** |
| Figure 3H  CD146 ELISA  E20 versus P2 | Unpaired t test | n=3 | p=0.0252, * |
| Figure 4A  mRNA CD146 | Unpaired t test | Ctrl pregnant mice n=3 (21 embryos)  Alc pregnant mice n=3 (21 embryos) | Ctrl vs Alc p=0.3092, ns |
| Figure 4C  mCD146 | Unpaired t test | Ctrl pregnant mice n=5 (32 embryos)  Alc pregnant mice n=5 (27 embryos) | Ctrl vs Alc p=0.4709, ns |
| Figure 4D  sCD146 | Unpaired t test | Ctrl pregnant mice n=5 (32 embryos)  Alc pregnant mice n=5 (27 embryos) | Ctrl vs Alc p<0.0001, **** |
| Figure 4E  mRNA CD146 | Unpaired t test | Ctrl pregnant mice n=3 (21 embryos)  Alc pregnant mice n=3 (21 embryos) | Ctrl vs Alc p=0.3772, ns |
| Figure 4G  mCD146 | Unpaired t test | Ctrl n=16 (from 4 pregnant mice)  Alc n=12 (from 3 pregnant mice) | Ctrl vs Alc p=0.6759, ns |
| Figure 4H  sCD146 | Unpaired t test | Ctrl n=16 (from 4 pregnant mice)  Alc n=12 (from 3 pregnant mice) | Ctrl vs Alc p=0.0233, * |
| Figure 4I  sCD146 ELISA | Two-way ANOVA  Tukey’s post test | Ctrl pregnant mice n= 3 (12 embryos)  Alc pregnant mice n= 3 (12 embryos) | ANOVA  Interaction (p=0.7085), ns  [F(1, 8)=0.1501]  Treatment effect p=0.0009, ***  [F(1, 8)=26.75]  Stages effect p=0.0022, **  [F(1, 8)=19.73]  Tukey’s post test at E20  Ctrl vs Alc, p=0.0388, *  Tukey’s post test at P2  Ctrl vs Alc, p=0.0183, * |
| Figure 5A  VEGF-R1 placenta | Unpaired t test | Ctrl pregnant mice n=3 (7 embryos)  Alc pregnant mice n=3 (7 embryos) | Ctrl vs Alc p=0.0290, * |
| Figure 5B  VEGF-R2 placenta | Unpaired t test | Ctrl pregnant mice n=5 (32 embryos)  Alc pregnant mice n=5 (27 embryos) | Ctrl vs Alc p=0.0399, * |
| Figure 5C  PSEN-1 placenta | Unpaired t test | Ctrl pregnant mice n=3 (11 embryos)  Alc pregnant mice n=3 (10 embryos) | Ctrl vs Alc p=0.0443, * |
| Figure 5D  Angiomotin placenta | Unpaired t test | Ctrl pregnant mice n=3 (11 embryos)  Alc pregnant mice n=3 (10 embryos) | Ctrl vs Alc p=0.0637, ns |
| Figure 5E  VEGF-R1  cortex | Unpaired t test | Ctrl pregnant mice n=5 (14 embryos)  Alc pregnant mice n=5 (14 embryos) | Ctrl vs Alc p=0.0417, * |
| Figure 5F  VEGF-R2  cortex | Unpaired t test | Ctrl pregnant mice n=3 (11 embryos)  Alc pregnant mice n=3 (11 embryos) | Ctrl vs Alc p=0.6836, ns |
| Figure 5G  PSEN-1  cortex | Unpaired t test | Ctrl pregnant mice n=4 (16 embryos)  Alc pregnant mice n=4 (16 embryos) | Ctrl vs Alc p=0.0276, * |
| Figure 5H  Angiomotin  cortex | Unpaired t test | Ctrl pregnant mice n=4 (18 embryos)  Alc pregnant mice n=4 (17 embryos) | Ctrl vs Alc p=0.1204, ns |
| Figure 6B  mCD146 repression | One-way ANOVA  Tukey’s post hoc test | n=3  (3 Ctrl, 3 Ctrl_ep_, 3 CD146-CRISPR) | ANOVA F 4.498  p=0.0404, *  Tukey’s p<0.05, *  Ctrl vs CD146-CRISPR  p=0.0359 |
| Figure 6F  Vessel distribution | Chi-square test | n=6  (6 Ctrl, 6 Ctrl_ep_, 6 CD146-CRISPR) | Ctrl vs CD146-CRISPR, Chi²=19.92, df=3  p=0.0002, ***  Ctrl vs Ctrl_ep_,  Chi²=2.525, df=3,  p=0.4708, ns |
| Figure 6G  Whole cortical vessel density | One-way ANOVA  Tukey’s post hoc test | n=6  (6 Ctrl, 6 Ctrl_ep_, 6 CD146-CRISPR) | ANOVA F 6.214  p=0.0108, *  Tukey’s p<0.05, *  Ctrl vs CD146-CRISPR  p=0.0320, *  Ctrl_ep_ vs CD146-CRISPR  p=0.0145, # |
| Figure 6H, left panel  Vessel density in superficial cortical layers | One-way ANOVA  Tukey’s post hoc test | n=6  (6 Ctrl, 6 Ctrl_ep_, 6 CD146-CRISPR) | ANOVA F 3.77  p=0.0472, *  Tukey’s p<0.05, *  Ctrl vs CD146-CRISPR  p=0.0422, *  Ctrl_ep_ vs CD146-CRISPR  p=0.1925, ns |
| Figure 6H, middle panel  Vessel density in deep cortical layers | One-way ANOVA  Tukey’s post hoc test | n=6  (6 Ctrl, 6 Ctrl_ep_, 6 CD146-CRISPR) | ANOVA F 1.63  p=0.2289, ns  Tukey’s p<0.05, *  Ctrl vs CD146-CRISPR  p=0.2478, ns  Ctrl_ep_ vs CD146-CRISPR  p=0.3536, ns |
| Figure 6H, right panel  Vessel density in superficial layers | One-way ANOVA  Tukey’s post hoc test | n=6  (6 Ctrl, 6 Ctrl_ep_, 6 CD146-CRISPR) | ANOVA F 7.994  p=0.0043, **  Tukey’s p<0.05, *  Ctrl vs CD146-CRISPR  p=0.0426, *  Ctrl_ep_ vs CD146-CRISPR  p=0.0037, ** |
| Figure 7H  Density of Olig2 cells in superficial cortical layers | One-way ANOVA  Tukey’s post hoc test | n=6  (6 Ctrl, 6 Ctrl_ep_, 6 CD146-CRISPR) | ANOVA F 5.01  p=0.0216, *  Tukey’s p<0.05, *  Ctrl vs CD146-CRISPR  p=0.0201, *  Ctrl_ep_ vs CD146-CRISPR  p=0.1018, ns |
| Figure 7I  Density of Olig2 cells in deep cortical layers | One-way ANOVA  Tukey’s post hoc test | n=6  (6 Ctrl, 6 Ctrl_ep_, 6 CD146-CRISPR) | ANOVA F 3.057  p=0.077, ns  Tukey’s p<0.05, *  Ctrl vs CD146-CRISPR  p=0.0666, ns  Ctrl_ep_ vs CD146-CRISPR  p=0.2985, ns |
| Figure 7J  Density of Olig2 cells in corpus callosum | One-way ANOVA  Tukey’s post hoc test | n=6  (6 Ctrl, 6 Ctrl_ep_, 6 CD146-CRISPR) | ANOVA F 5.425  p=0.0169, *  Tukey’s p<0.05, *  Ctrl vs CD146-CRISPR  p=0.013, *  Ctrl_ep_ vs CD146-CRISPR  p=0.1999, ns |
| Figure 7K  Percentage of vessel-associated Olig2 cells in cortical layers | One-way ANOVA  Tukey’s post hoc test | n=6  (6 Ctrl, 6 Ctrl_ep_, 6 CD146-CRISPR) | ANOVA F 0.1169  p=0.8902, ns  Tukey’s p=0.05, ns  Ctrl vs CD146-CRISPR  p=0.9535, ns  Ctrl_ep_ vs CD146-CRISPR  p=9885, ns |
| Figure 8A  Regression analysis of cortical Olig2 and microvessel densities | Correlation test | n=9 paired values  (3 Ctrl, 3 Ctrl_ep_, 3 CD146-CRISPR) | R² 0.6256  p=0.0111, * |
| Figure 8B  Regression analysis of cortical Olig2 and microvessel densities in SL, DL and CC | Correlation test | n=9 paired values  (3 XY pairs SL, 3 XY pairs DL and 3 XY pairs CC) | SL  R² 0.9983  p=0.0265, *  DL  R² 0.9461  p=0.1492, ns  CC  R² 0.5081  p=0.4948, ns |
| Suppl Figure 1  Effect of alcohol on placental sCD146 from female and male fetuses at E20 | Unpaired t test | Ctrl placentas from female fetuses, n=7  Alc placentas from female fetuses, n=5  Ctrl placentas from male fetuses, n=7  Alc placentas from male fetuses, n=8 | Ctrl vs Alc  p=0.0406, *  Ctrl vs Alc  p=0.0211, * |
| Suppl Figure 2  Regression analysis of sCD146 between placenta, fetal blood and fetal brain | Unpaired t test | n=3 | Placenta-blood slope (Ctrl)  vs  Placenta-blood slope (Alc)  p=0.1230, ns  Placenta-brain slope (Ctrl)  vs  Placenta-brain slope (Alc)  p=0.0002, *** |
