# Supplementary figures and images for: Expression of placental CD146 is dysregulated by prenatal alcohol exposure and contributes in cortical vasculature development and positioning of vessel-associated oligodendrocytes
Source: Front Cell Neurosci. 2024 Jan 10;17:1294746. doi: 10.3389/fncel.2023.1294746 (PMC10806802; doi:10.3389/fncel.2023.1294746)

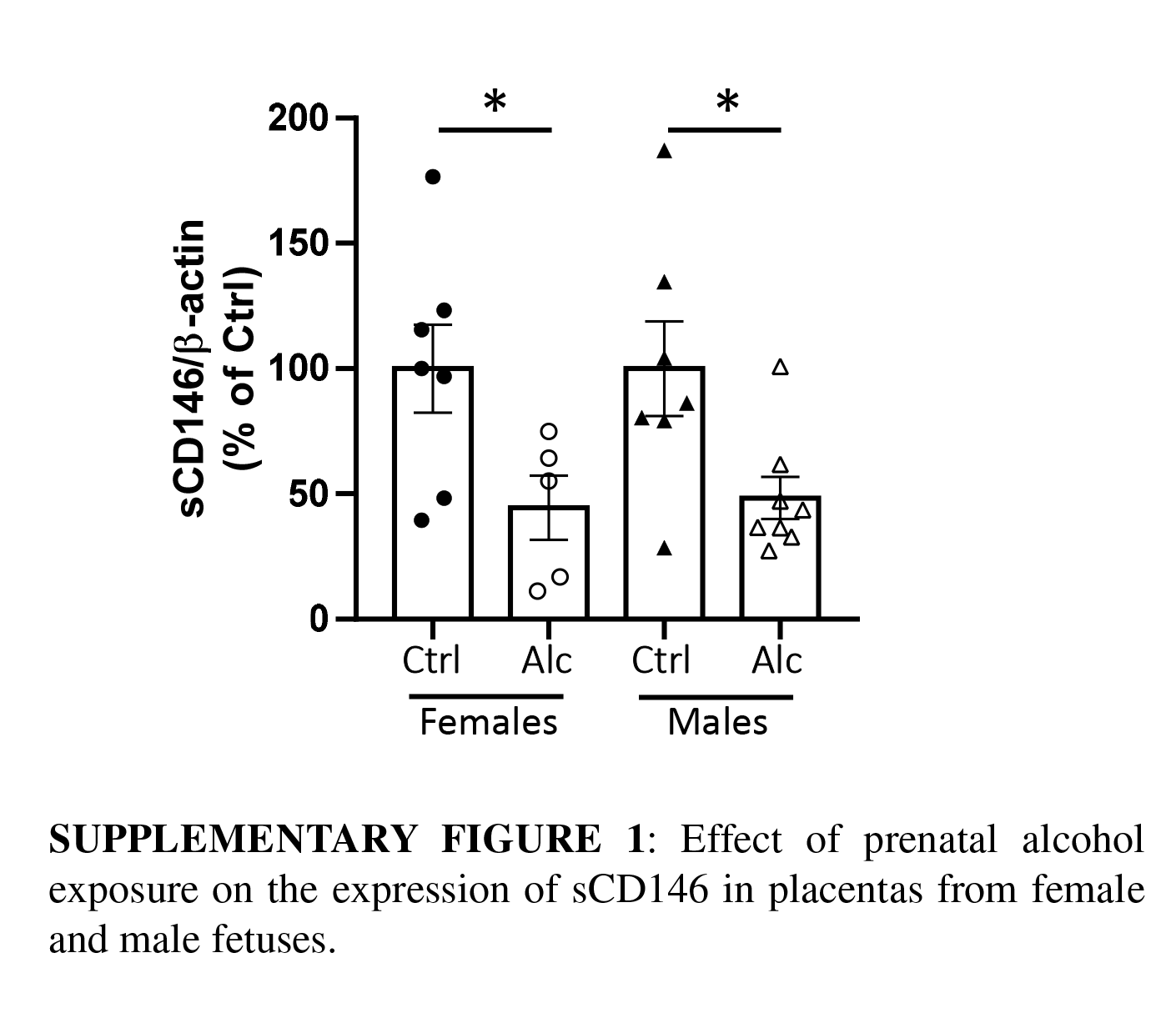

Supplement: Supplementary file 4 [file Image_1.TIF]

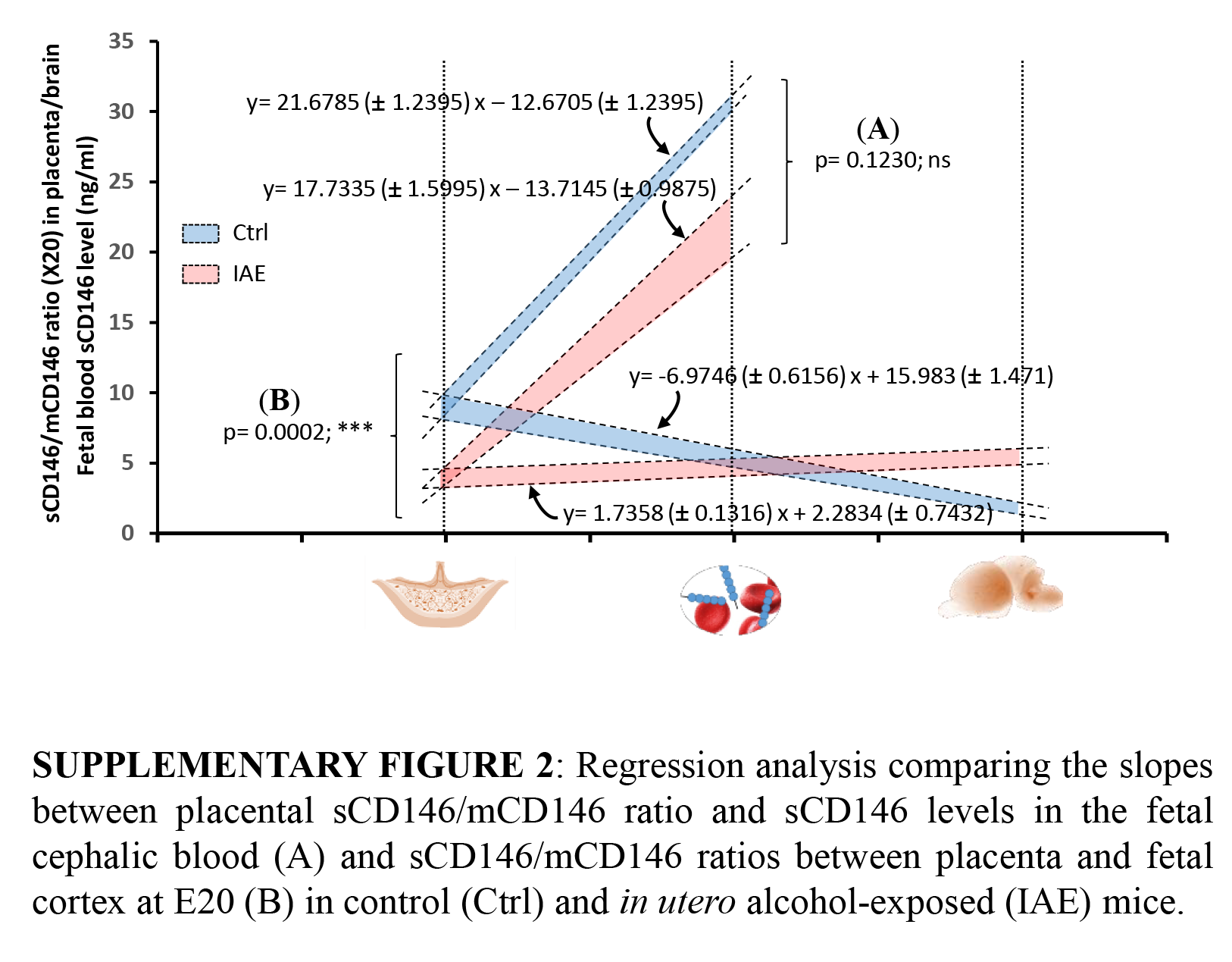

Supplement: Supplementary file 5 [file Image_2.TIF]

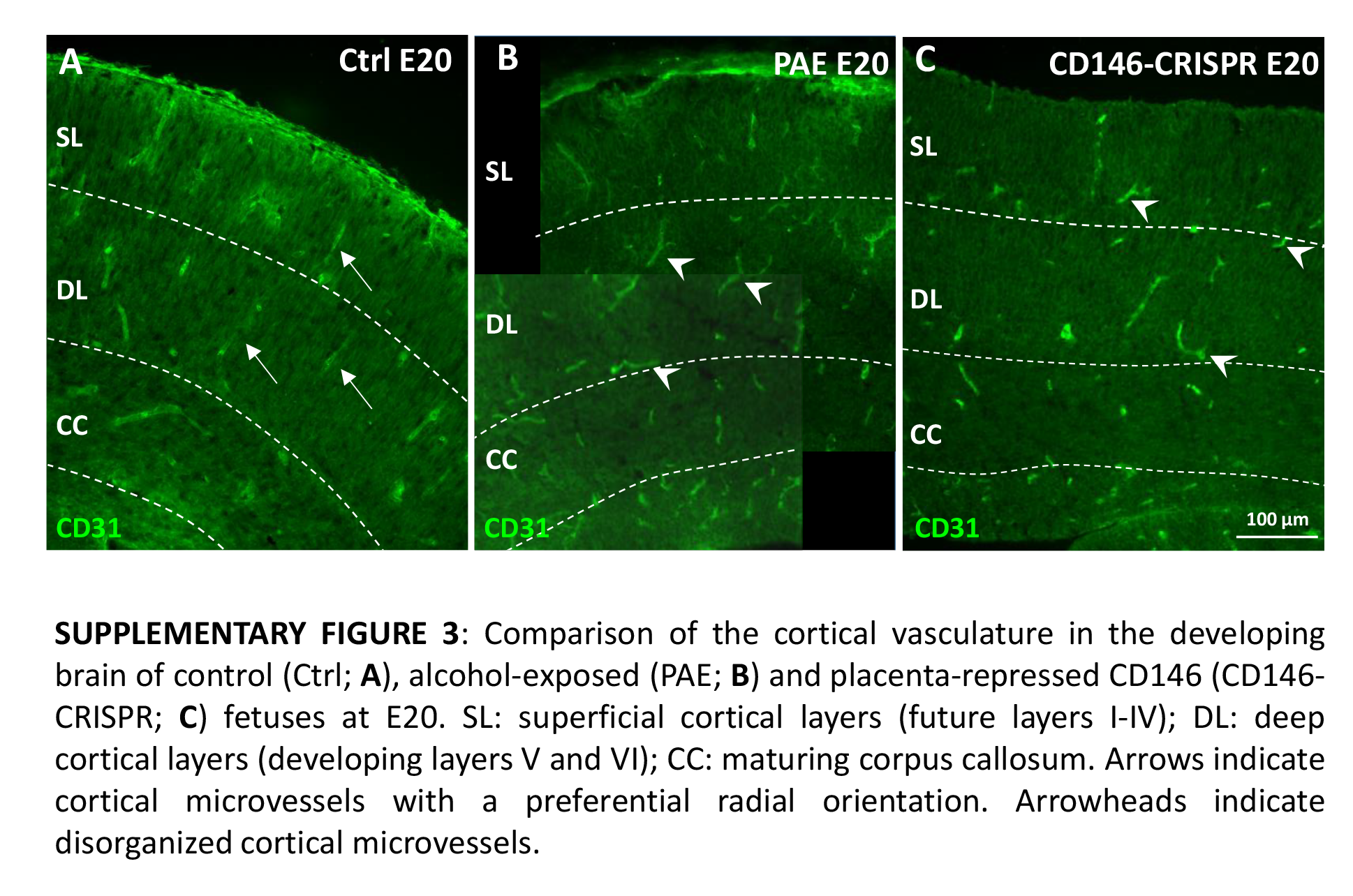

Supplement: Supplementary file 6 [file Image_3.TIF]

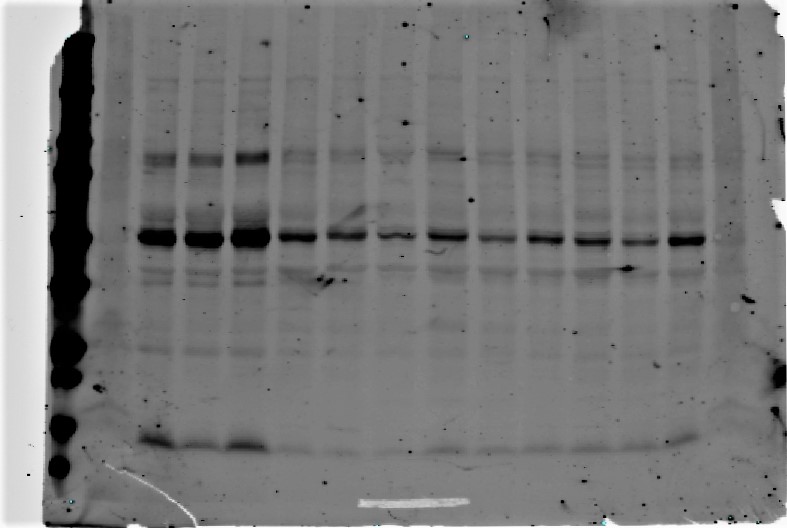

Supplement: Supplementary file 9 [file Data_Sheet_1.ZIP › original blot fig 1F mCD146 and sCD146 human placenta.tif]

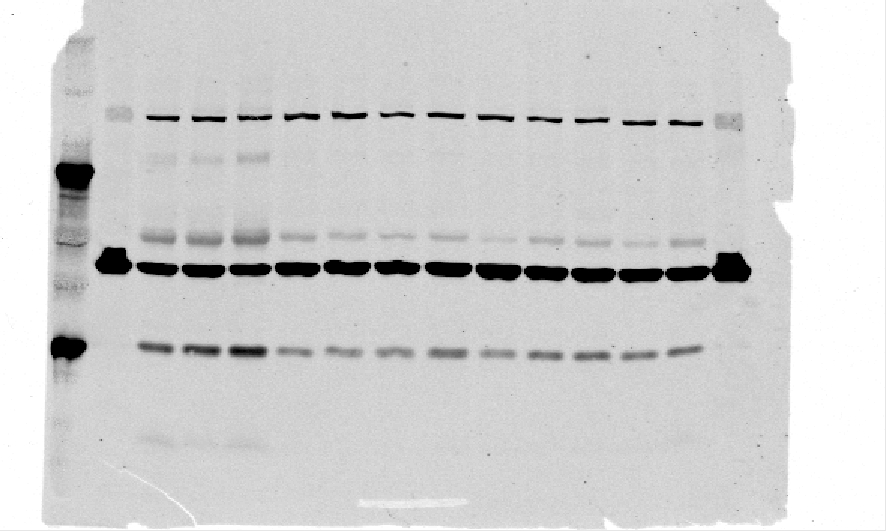

Supplement: Supplementary file 9 [file Data_Sheet_1.ZIP › original blot fig 1F vimentin human placenta.tif]

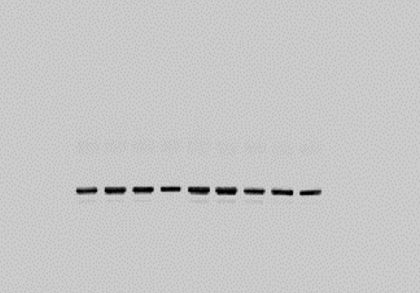

Supplement: Supplementary file 9 [file Data_Sheet_1.ZIP › original blot fig 2B actin mouse placenta.tif]

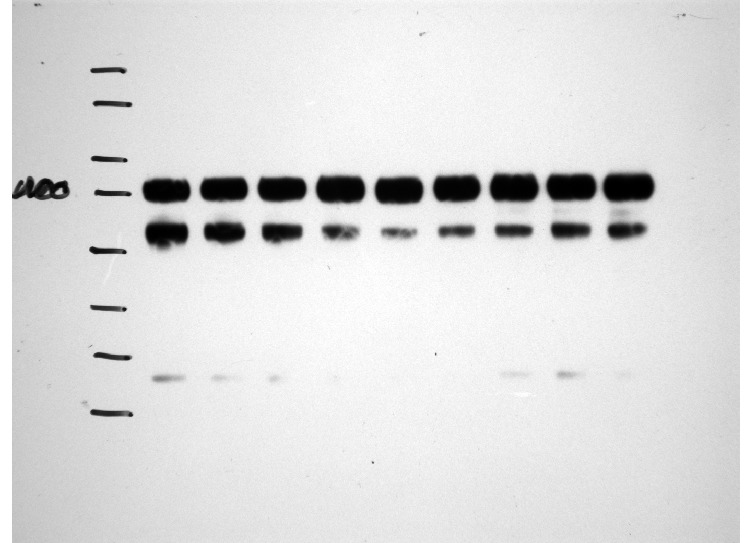

Supplement: Supplementary file 9 [file Data_Sheet_1.ZIP › original blot fig 2B mCD146 and sCD146 mouse placenta.tif]

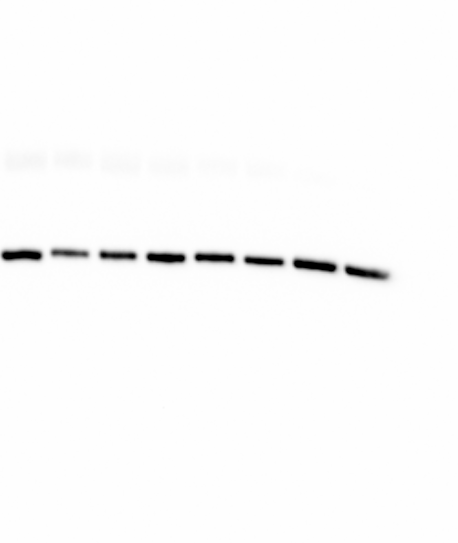

Supplement: Supplementary file 9 [file Data_Sheet_1.ZIP › original blot fig 3B actin mouse cortex.tif]

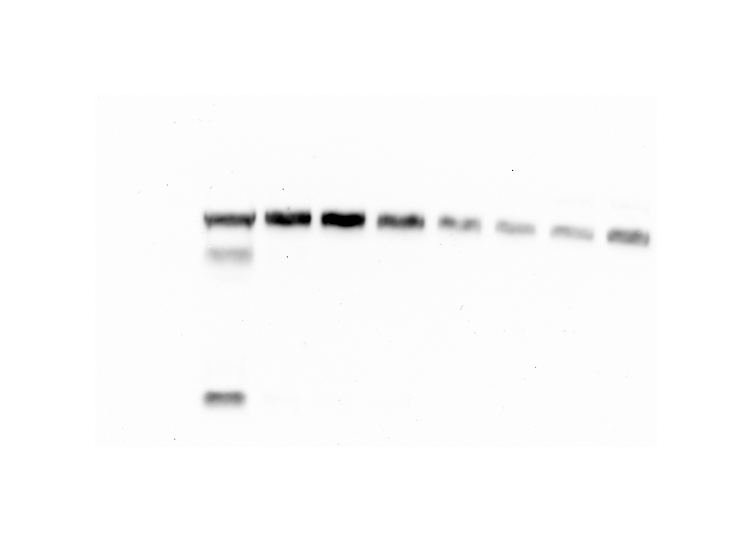

Supplement: Supplementary file 9 [file Data_Sheet_1.ZIP › original blot fig 3B mCD146 and sCD146 mouse cortex.tif]

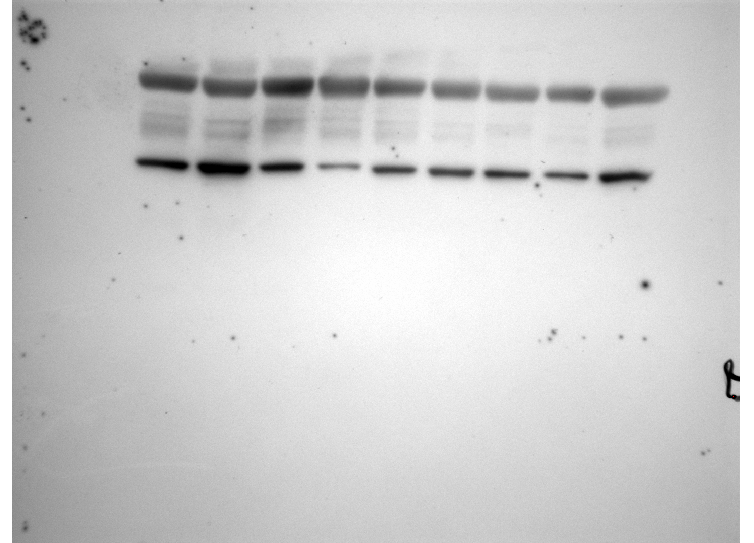

Supplement: Supplementary file 9 [file Data_Sheet_1.ZIP › original blot fig 4B actin mouse placenta.tif]

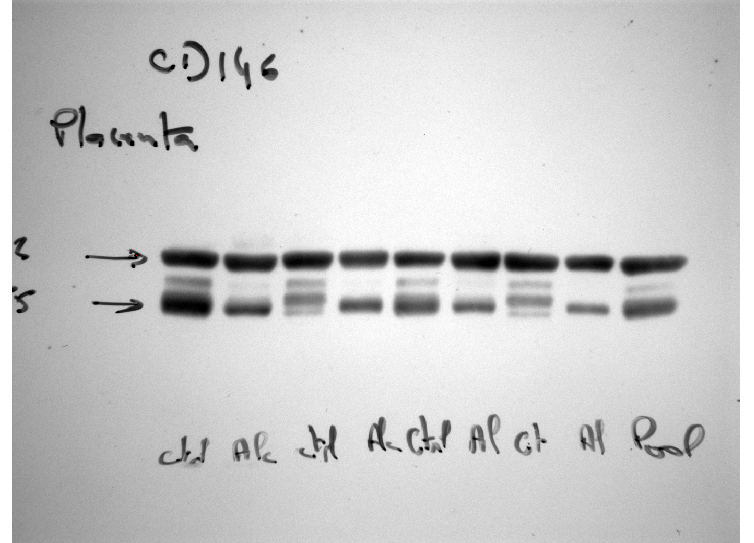

Supplement: Supplementary file 9 [file Data_Sheet_1.ZIP › original blot fig 4B mCD146 and sCD146 mouse placenta.tif]

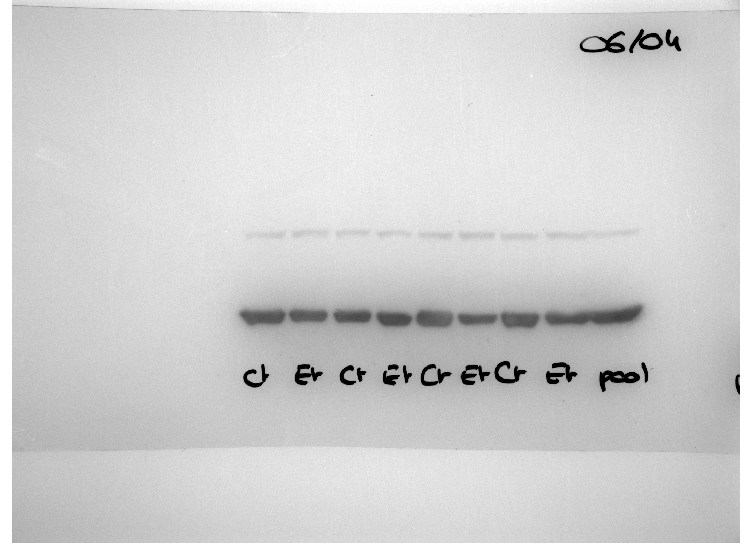

Supplement: Supplementary file 9 [file Data_Sheet_1.ZIP › original blot fig 4F actin mouse cortex.tif]

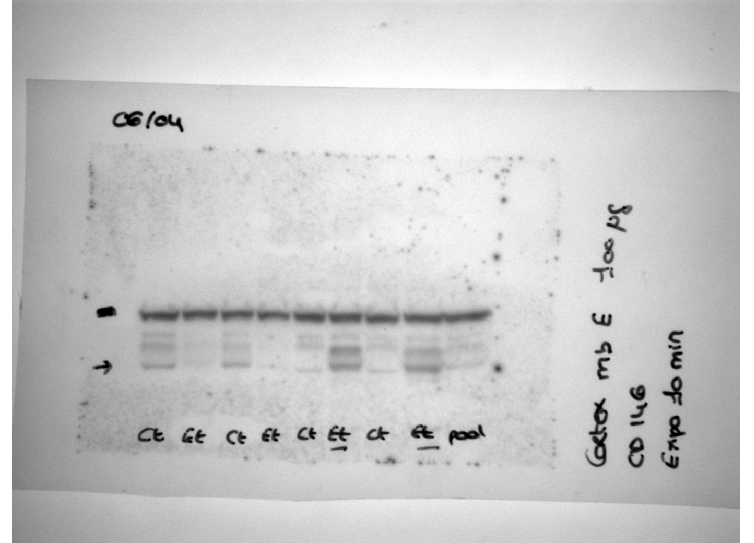

Supplement: Supplementary file 9 [file Data_Sheet_1.ZIP › original blot fig 4F mCD146 and sCD146 mouse cortex.tif]

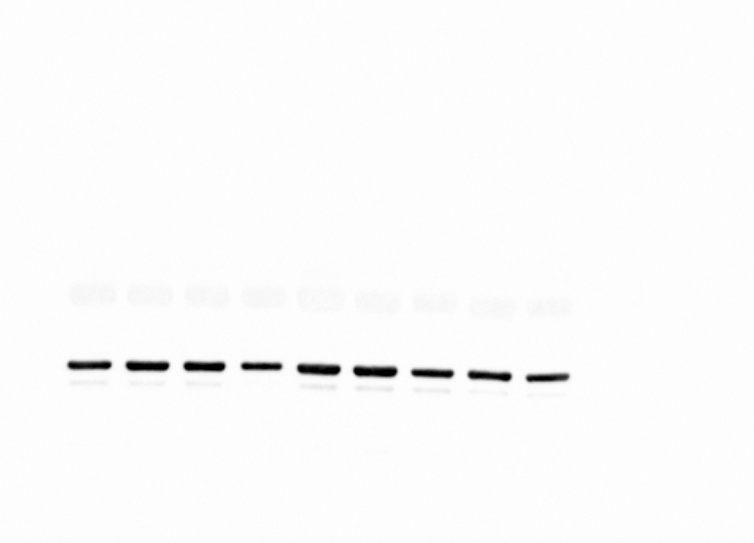

Supplement: Supplementary file 9 [file Data_Sheet_1.ZIP › original blot fig 5A actin mouse placenta.tif]

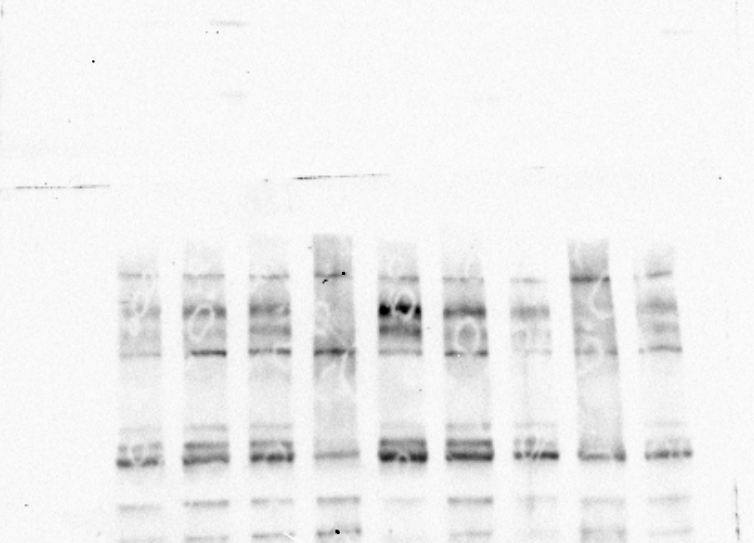

Supplement: Supplementary file 9 [file Data_Sheet_1.ZIP › original blot fig 5A VEGF-R1 mouse placenta.tif]

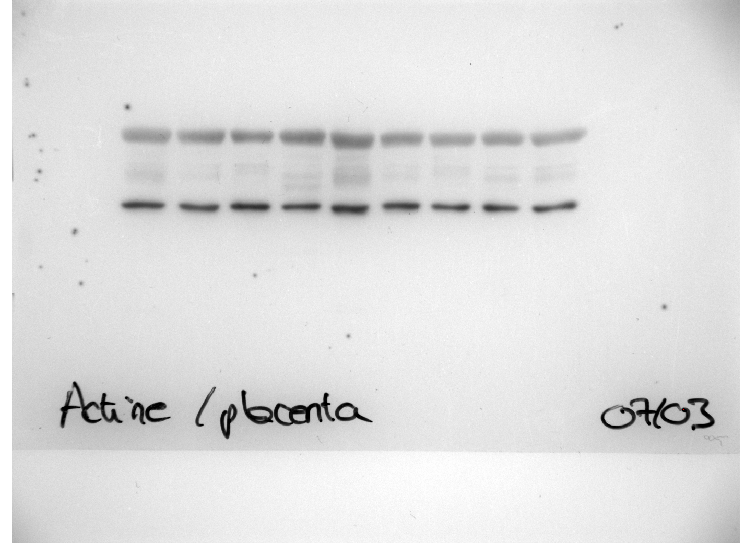

Supplement: Supplementary file 9 [file Data_Sheet_1.ZIP › original blot fig 5B actin mouse placenta.tif]

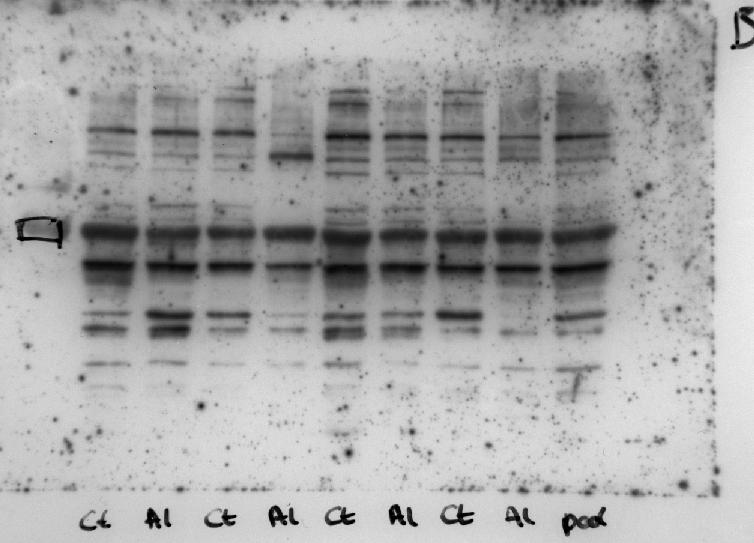

Supplement: Supplementary file 9 [file Data_Sheet_1.ZIP › original blot fig 5B VEGF-R2 mouse placenta.tif]

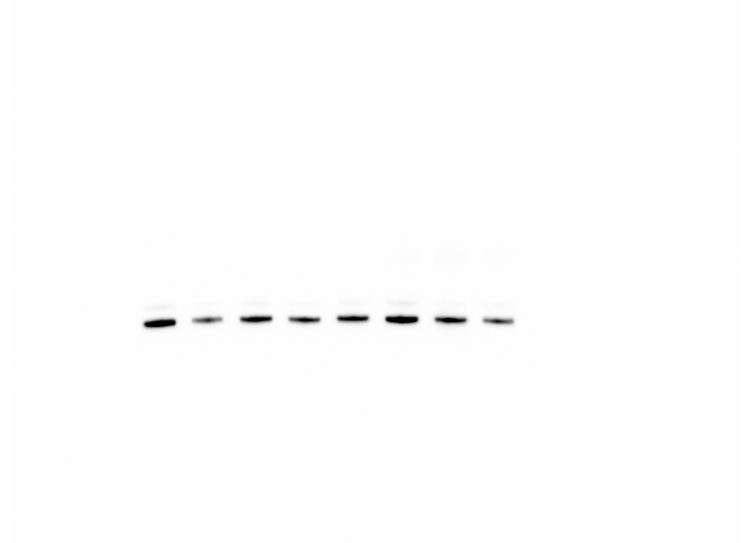

Supplement: Supplementary file 9 [file Data_Sheet_1.ZIP › original blot fig 5C actin mouse placenta.tif]

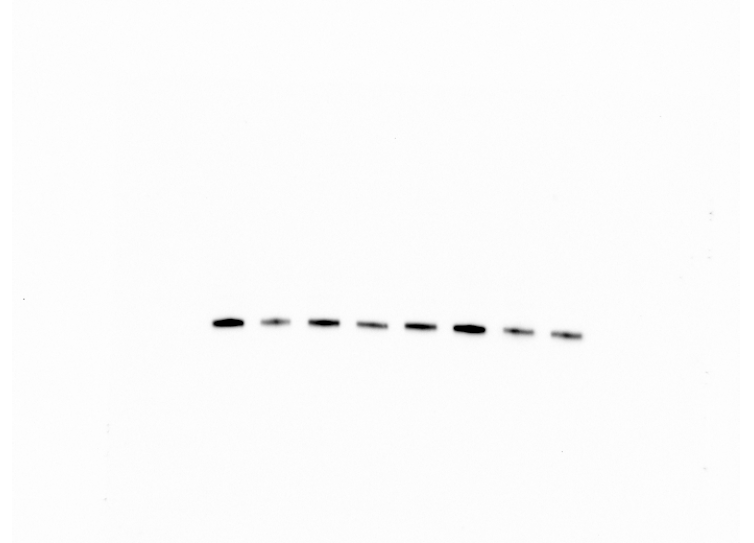

Supplement: Supplementary file 9 [file Data_Sheet_1.ZIP › original blot fig 5C PSEN-1 mouse placenta.tif]

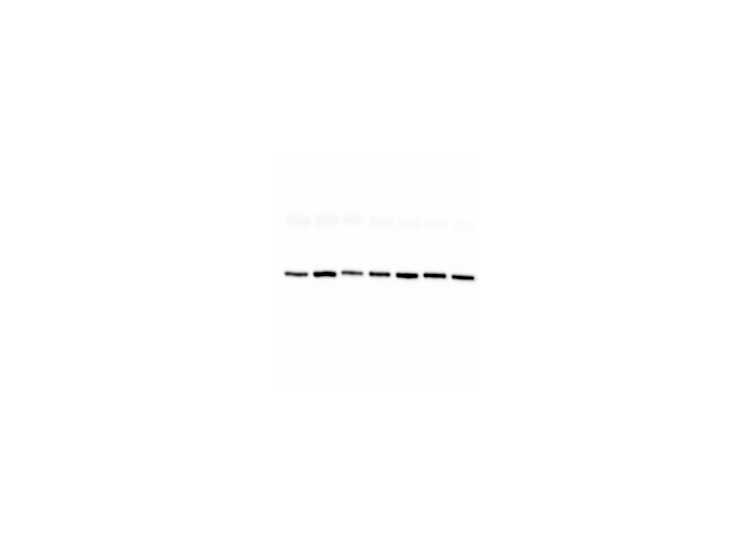

Supplement: Supplementary file 9 [file Data_Sheet_1.ZIP › original blot fig 5D actin mouse placenta.tif]

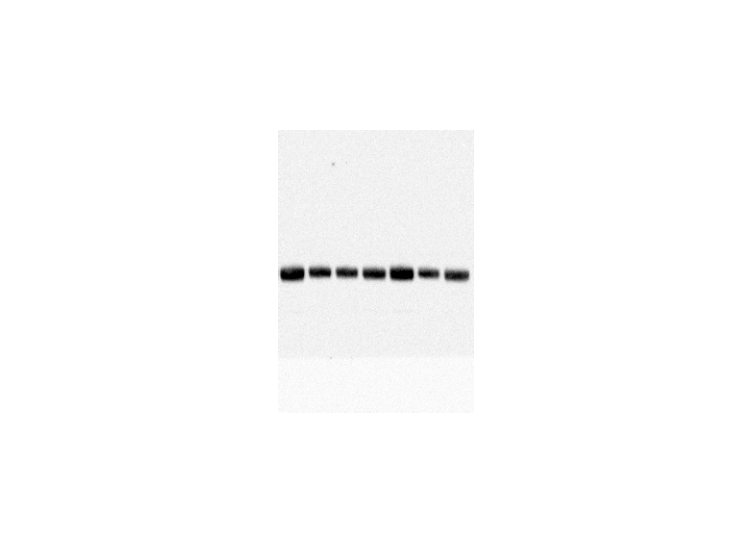

Supplement: Supplementary file 9 [file Data_Sheet_1.ZIP › original blot fig 5D angiomotin mouse placenta.tif]

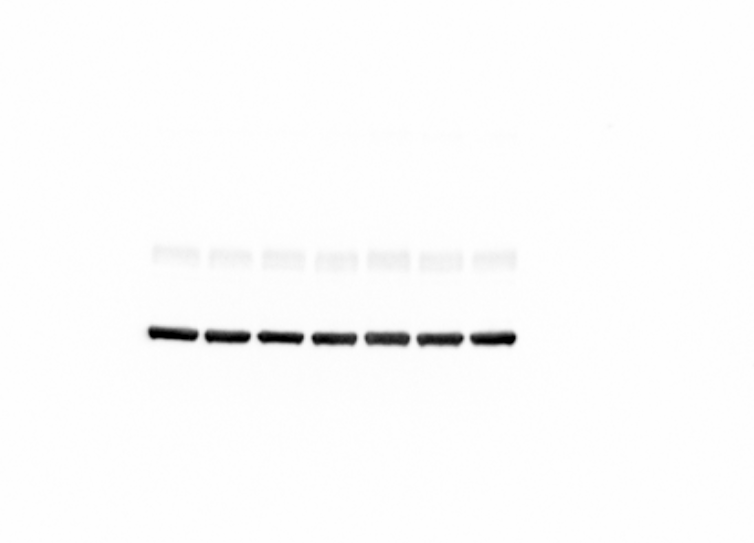

Supplement: Supplementary file 9 [file Data_Sheet_1.ZIP › original blot fig 5E actin mouse cortex.tif]

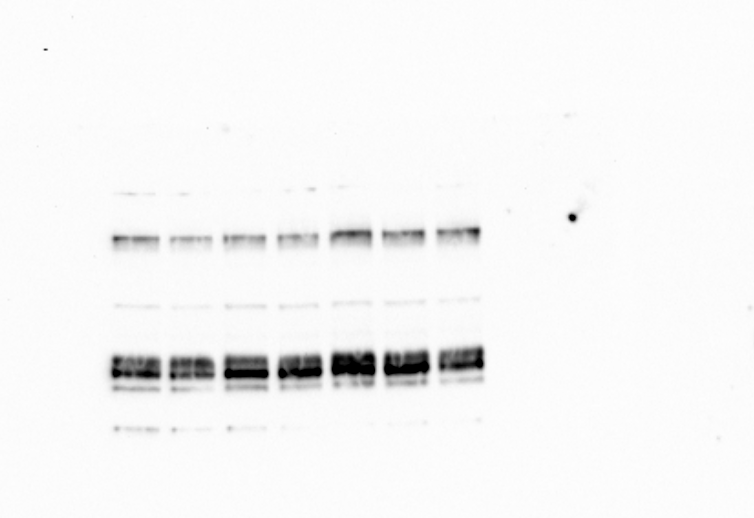

Supplement: Supplementary file 9 [file Data_Sheet_1.ZIP › original blot fig 5E VEGF-R1 mouse cortex.tif]

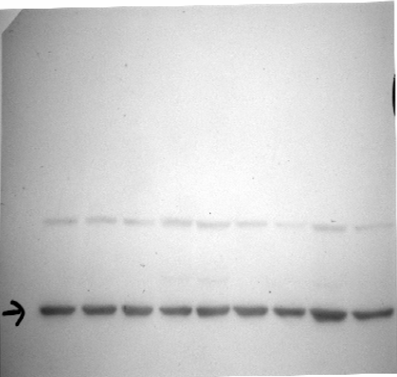

Supplement: Supplementary file 9 [file Data_Sheet_1.ZIP › original blot fig 5F actin mouse cortex.tif]

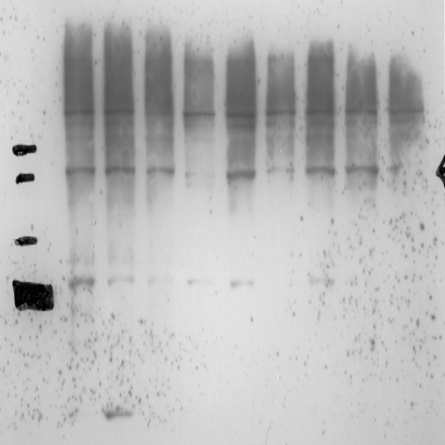

Supplement: Supplementary file 9 [file Data_Sheet_1.ZIP › original blot fig 5F VEGF-R2 mouse cortex.tif]

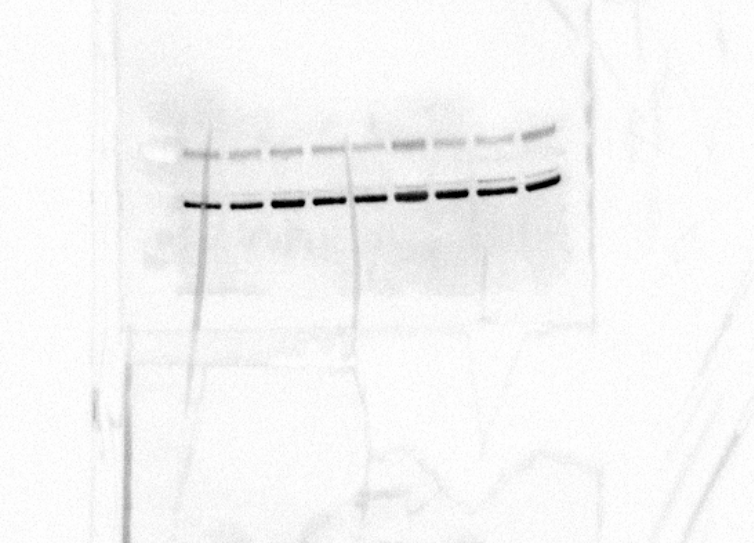

Supplement: Supplementary file 9 [file Data_Sheet_1.ZIP › original blot fig 5G actin mouse cortex.tif]

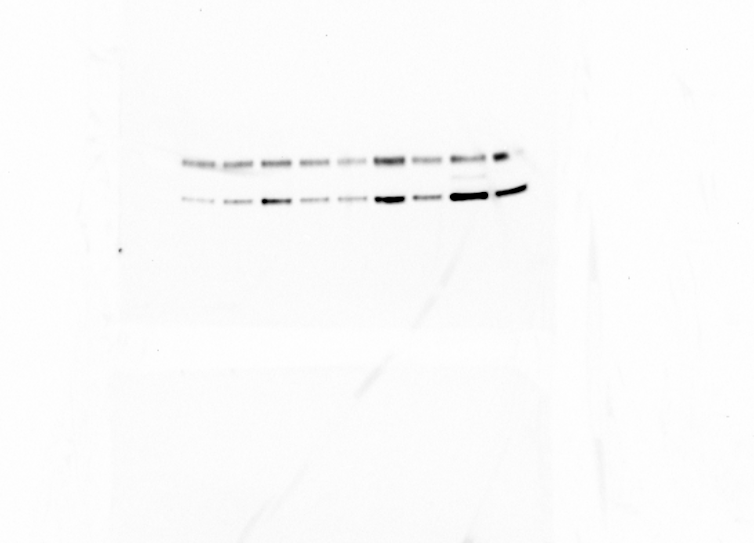

Supplement: Supplementary file 9 [file Data_Sheet_1.ZIP › original blot fig 5G PSEN-1 mouse cortex.tif]

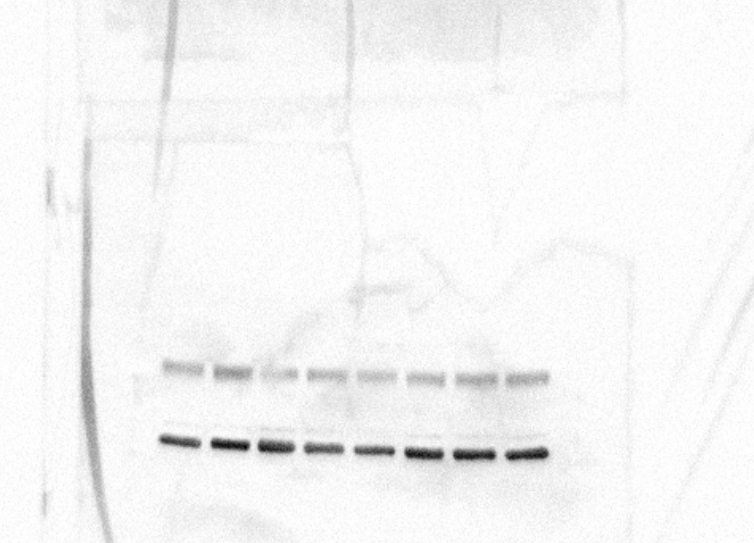

Supplement: Supplementary file 9 [file Data_Sheet_1.ZIP › original blot fig 5H actin mouse cortex.tif]

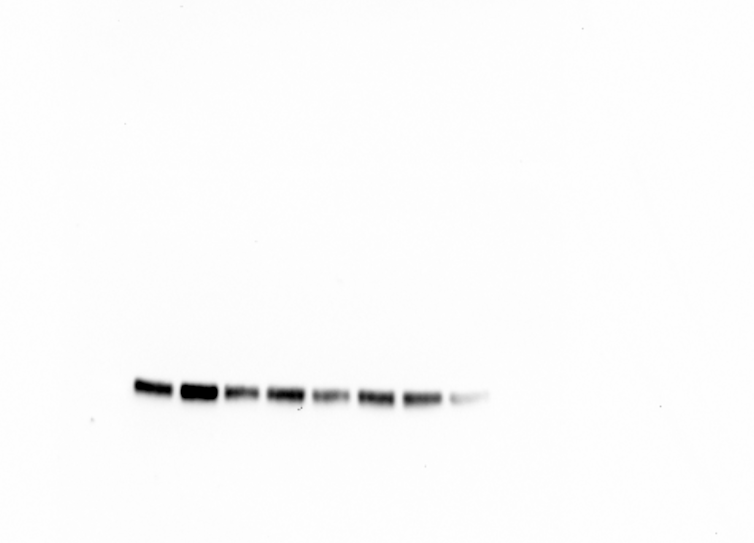

Supplement: Supplementary file 9 [file Data_Sheet_1.ZIP › original blot fig 5H angiomotin mouse cortex.tif]

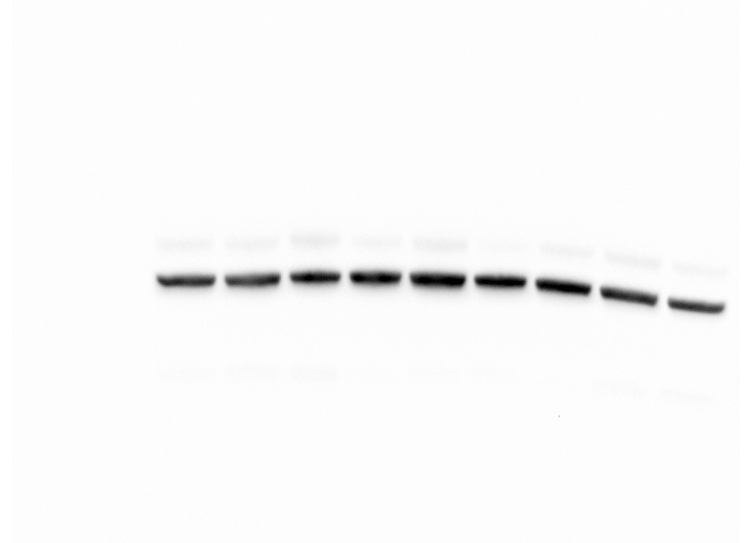

Supplement: Supplementary file 9 [file Data_Sheet_1.ZIP › original blot fig 6B actin mouse placenta.tif]

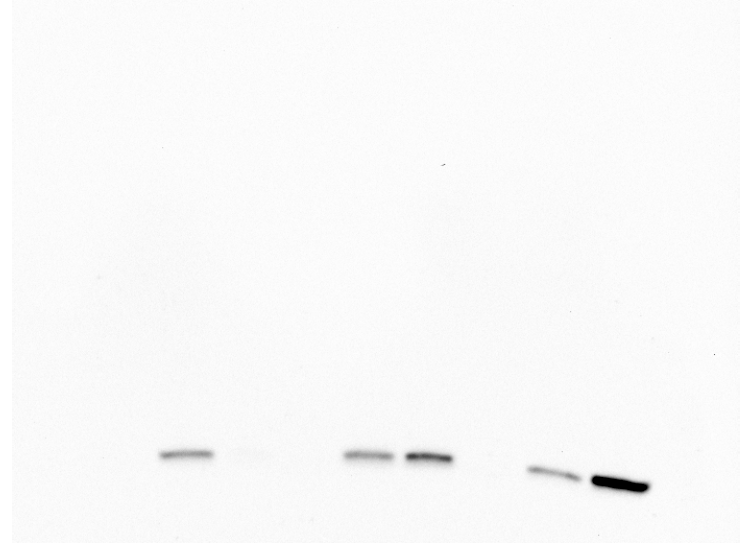

Supplement: Supplementary file 9 [file Data_Sheet_1.ZIP › original blot fig 6B GFP mouse placenta.tif]

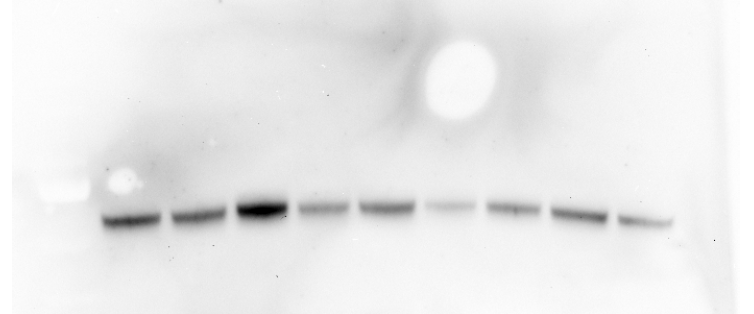

Supplement: Supplementary file 9 [file Data_Sheet_1.ZIP › original blot fig 6B mCD146 mouse placenta.tif]
